# Multiple *Nf1* Schwann cell populations reprogram the plexiform neurofibroma tumor microenvironment

**DOI:** 10.1172/jci.insight.154513

**Published:** 2022-09-22

**Authors:** Leah J. Kershner, Kwangmin Choi, Jianqiang Wu, Xiyuan Zhang, Melissa Perrino, Nathan Salomonis, Jack F. Shern, Nancy Ratner

**Affiliations:** 1 Division of Experimental Hematology and Cancer Biology, Cincinnati Children’s Hospital Medical Center, University of Cincinnati, Cincinnati, Ohio, USA.; 2 Pediatric Oncology Branch, National Cancer Institute, Bethesda, Maryland, USA.; 3 Division of Biomedical Informatics, and; 4 Departments of Pediatrics and Bioinformatics, University of Cincinnati, Cincinnati, Ohio, USA.

**Keywords:** Cell Biology, Oncology, Expression profiling, Genetic diseases, NF-kappaB

## Abstract

To define alterations early in tumor formation, we studied nerve tumors in neurofibromatosis 1 (NF1), a tumor predisposition syndrome. Affected individuals develop neurofibromas, benign tumors driven by *NF1* loss in Schwann cells (SCs). By comparing normal nerve cells to plexiform neurofibroma (PN) cells using single-cell and bulk RNA sequencing, we identified changes in 5 SC populations, including a de novo SC progenitor–like (SCP-like) population. Long after *Nf1* loss, SC populations developed PN-specific expression of *Dcn*, *Postn*, and *Cd74*, with sustained expression of the injury response gene *Postn* and showed dramatic expansion of immune and stromal cell populations; in corresponding human PNs, the immune and stromal cells comprised 90% of cells. Comparisons between injury-related and tumor monocytes/macrophages support early monocyte recruitment and aberrant macrophage differentiation. Cross-species analysis verified each SC population and unique conserved patterns of predicted cell-cell communication in each SC population. This analysis identified PROS1-AXL, FGF-FGFR, and MIF-CD74 and its effector pathway NF-κB as deregulated in *NF1* SC populations, including SCP-like cells predicted to influence other types of SCs, stromal cells, and/or immune cells in mouse and human. These findings highlight remarkable changes in multiple types of SCs and identify therapeutic targets for PN.

## Introduction

Neurofibromatosis type 1 (NF1) is a common genetic human disorder with a frequency of approximately 1:2500 worldwide (1). Plexiform neurofibromas (PNs) occur in 30%–50% of NF1 patients and arise in peripheral nerve bundles, frequently causing substantial morbidity, including pain, neurologic deficit, and motor dysfunction (2). PNs are considered benign, yet no cure exists for PN apart from surgical removal, which is often impossible due to their large size, association with critical anatomic structures, and nerve integration.

*NF1* encodes neurofibromin, a GTPase-activating protein (GAP) that augments the intrinsic GAP activity of RAS proteins; thus, mutation or loss of *NF1* activates signaling through the RAS pathway (3). In a PN genetically engineered mouse model (GEMM; *Dhh-Cre*;*Nf1^fl/fl^*), small-molecule targeting of the RAS/MAPK pathway (e.g., MEK inhibitors) caused tumor shrinkage (4, 5). This result predicted the success of the MEK inhibitor selumetinib in clinical trials, which showed PN shrinkage of 20% or greater in 70% of patients with inoperable PNs (5, 6) and led to FDA approval. Importantly, the successful translation of preclinical findings into the clinic validated and underscored the utility of the PN GEMM for modeling the human disease. While exciting, selumetinib caused maximal PN shrinkage of 50%, and sustained drug administration was required to maintain shrinkage. This resistance may be due to cell autonomous changes in tumor cells and/or to effects on the tumor microenvironment.

The tumorigenic cells in neurofibromas are nerve glial cells called Schwann cells (SCs), reported to comprise 40%–80% of human neurofibroma cells (7). Loss-of-function mutations in both *NF1* alleles are detected in neurofibroma SCs but not in other neurofibroma cell types (8, 9). PN GEMMs in which *NF1* loss is specific to SCs develop PNs (10, 11) that, like human PNs, contain numerous cell types (12). Notably, while macrophages are present in low abundance in normal nerve, 30% of PN cells are Iba1^+^CD11b^+^F4/80^+^ monocytes/macrophages that may play roles in PN initiation and growth (13–15). Neurofibromas also contain a network of nerve CD34^+^ fibroblasts, believed to manufacture most of the collagen-rich matrix that accounts for much of PN dry weight (7, 16). Minor PN cell populations of dendritic cells (DCs) and T cells are implicated in tumor initiation (17). Mast cells are present in neurofibromas, although their genetic ablation does not affect tumor formation (18). None of these immune or stromal cell types are well characterized.

SCs and/or their progenitors are crucial for neurofibroma formation. In SC development, embryonic neural crest cells that contact peripheral neuron axons differentiate into SC precursors (SCPs), and then immature SCs (19). SCPs have properties of stem/progenitor cells, which can differentiate along multiple lineages and self-renew in vitro. SCPs are present in developing nerve and in postnatal rodent dorsal root ganglia (DRGs) but not in adult nerve (20). In GEMM, *Nf1* loss of function in nerve glial cells at the SCP stage is sufficient to cause PNs (10, 11, 21, 22). Mouse and human PNs contain rare (1%–2%) EGFR^+^ cells that express the SC marker S100 (23). Cells positive for the SC lineage marker P75NGFR and EGFR sorted from human tumors have properties of stem/progenitor cells, including self-renewal and multiple-lineage differentiation (24). Supporting the idea that PN SCP-like cells are tumor-initiating cells, *Nf1*-mutant embryonic SCPs and P75NGFR^+^EGFR^+^ SCP-like cells from PNs form neurofibromas on in vivo transplantation (15, 24, 25). Characterization of these cells is critical for mechanistic understanding of tumorigenesis.

SCs that attach to neuronal cell bodies form satellite glial cells (SGCs) (26). Other SCs actively sort large diameter neural axons to form promyelinating SCs and then myelinating SCs. In contrast, SCs associated with groups of small diameter axons (<1 μm) form nonmyelinating SCs (NMSCs) called Remak cells, likely through a transitional SC intermediate (27). In Remak bundles a single NMSC packages 6–20 small diameter axons (28). In PN mouse models, mature NMSCs progressively dissociate from nerve axons and have been proposed as a source of PNs (29, 30). Similarly, in human PNs, NMSCs ensheath 0–2 axons (31). How each SC population is affected by loss of *NF1* and/or whether specific SC populations modulate functions of PN immune/stromal cells is unknown.

To define the PN transcriptome at single-cell resolution, we used single-cell RNA sequencing (scRNA-seq) in the *Dhh-Cre*;*Nf1^fl/fl^* PN GEMM and human clinical samples. The resolution of these experiments allowed the identification of extensive changes in PN cell populations that occur over time in the *Dhh-Cre*;*Nf1^fl/fl^* PN model and the discovery of similar cellular populations in human PNs. This data set allowed us to comprehensively define the cell-cell communication predicted to occur, in both species, between tumor SCs and immune/stromal cells. Finally, we identify several pathways that may be targets for therapy.

## Results

**Figure 1:**
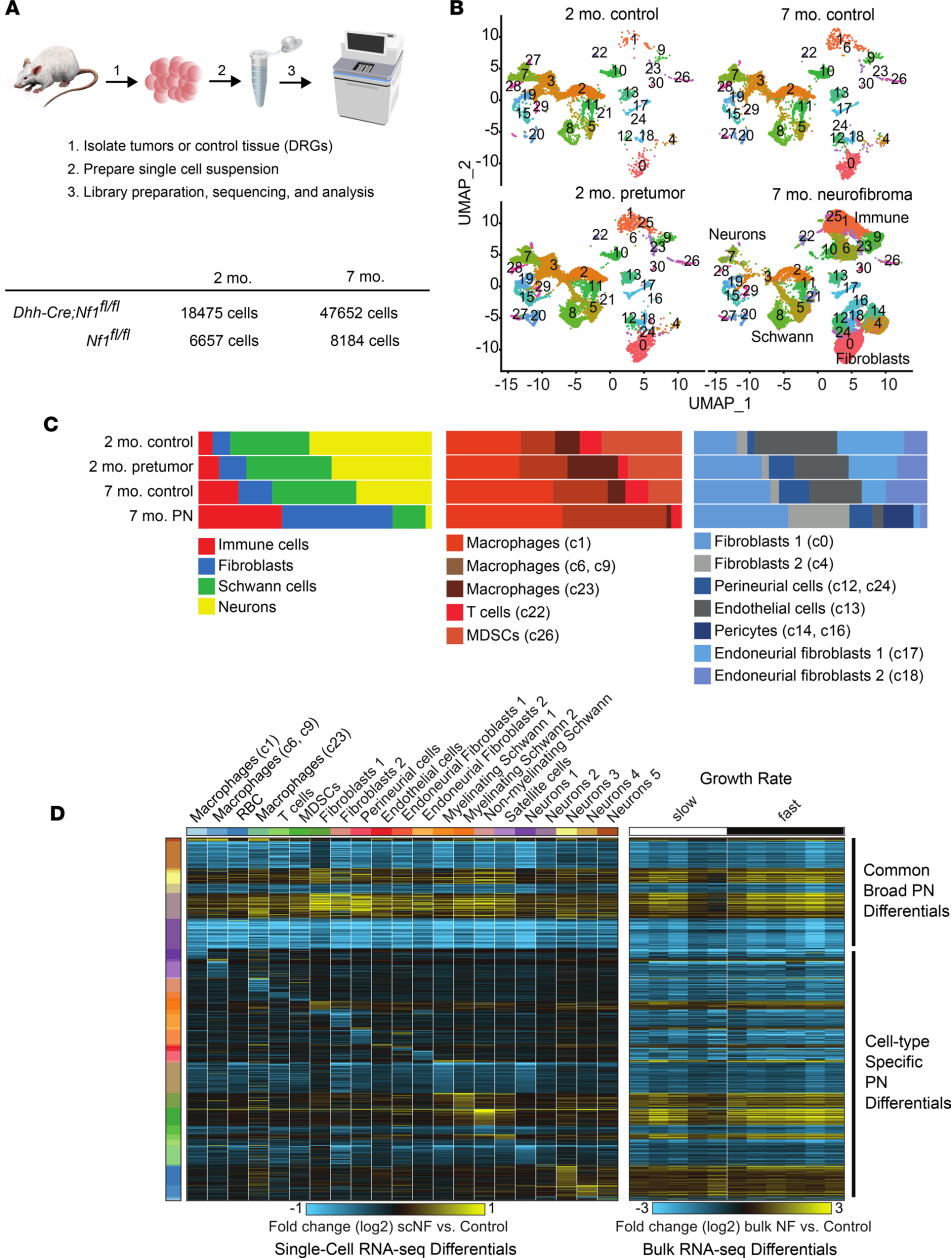
Immune and stromal cell increases in PNs occur long after SC loss of Nf1. (**A**) Schematic of the analysis of control and PN cells by scRNA-seq showing analyzed cell numbers. (**B**) UMAPs of PNs (7-month *Dhh*-*Cre;Nf1^fl/fl^*), pretumor (2-month *Dhh*-*Cre;Nf1^fl/fl^*), and corresponding 7-month and 2-month *Nf1^fl/fl^* littermate controls, generated using Seurat. (**C**) Cell type frequencies across sample types showing all cell types (left); immune cells (middle), and stromal cells (right), showing enrichment of immune and stromal cell clusters in PNs. UMAP cluster numbers (from **B**) are shown in parentheses for each annotated cell type. (**D**) Left: Heatmap showing scRNA-seq gene fold changes in 7-month PN cells versus controls, generated using cellHarmony. Right: Differential expression of the same genes in bulk RNA-seq, analyzed as fold changes in 7-month PNs versus controls. The changes were independent of PN growth rate (fast/slow). Broad patterns of differential gene expression across cell types and cell type specific changes are evident.

To characterize transcriptional programs activated by loss of *Nf1* in the SC lineage we performed scRNA-seq, using cells from the *Dhh-Cre*;*Nf1^fl/fl^* GEMM. In this model, PNs primarily form around cervical DRGs, so as controls we used normal DRGs, with associated nerve roots and proximal nerve trunks from littermates. We isolated cells at 2 months old (before PNs form in mutants) and at 7 months old (when PNs are present in all mutants) (Figure 1A). We performed dimensionality reduction, clustering, and sample integration to define 31 unique cell populations in PNs and controls (Figure 1B); 25 distinct cell populations remained after requiring more than 3 unique marker genes/cluster and no unique clusters had a doublet percentage of greater than 15% using Scrublet (32). Notably, even though *Dhh-Cre* causes loss of *Nf1* in SCs at embryonic day 12.5 (E12.5), cell population changes were robust only later, in 7-month PNs. In total, 5 immune cell populations and 7 stromal cell clusters were present (Figure 1C). Significant heterogeneity of, and increase in proportions of, immune and stromal cell populations developed in PNs over time, as compared either with littermate 7-month wild-type controls or with 2-month mutants (*Dhh*-*Cre*;*Nf1^fl/fl^* [pretumor]).

### Mouse PN immune and stromal cells.

Immune and stromal cell populations included 2 populations: Macrophages 1 (c1: *C1qa*, *C1qb*, *C3aR1*, *Cd68*, *Csf1r*, *Tlr2*, and *Cd86*) and 2 (c6 + c9: *Ccr7*, *Csf2rb*, *Il1b*, and *Cd74*) expanded in PNs as a percentage of total tumor cells (Figure 1C). The c6 macrophage population that appeared de novo (c6: *CD36*, *Il1b*, *P2ry10*, and *Cd83*) did not contain more than 3 unique markers. DCs clustered with Macrophage c1; they expressed *Cd14*, *Ctss*, *Lgals3*, *Ms4a7*, *H2-Eb1*, *Cxcl16*, *Stamf7*, *Adgre1*, *Trem2*, and *Retla*. Mast cells, characterized by expression of *Cma1* and *Mcpt4* in peripheral nerves (33), clustered alongside Macrophages c9.

We also identified genes characteristic of putative myeloid-derived suppressor cells (MDSCs) (*Aspv1*, *Clec4d*, *Clec43*, *Ccr2*, *Cxcr2*, *Il1b*, and *Csf3r*) (34), which have not previously been described to the best of our knowledge in PNs. The MDSC cluster accounted for less than 1% of total immune cells in PNs, was enriched in *Il1b*, and uniquely expressed *Clec4d*, *Clec4e*, and *Csf3r* (Supplemental Table 2; supplemental material available online with this article; https://doi.org/10.1172/jci.insight.154513DS1). *S100a9*, encoding a protein with key roles in the regulation of inflammatory processes and immune response, was the top marker of this cluster; *S100a9* is typically expressed at lower levels in monocytes/macrophages and DCs. We also confirmed the presence of T cells, with proportions consistent across all conditions (4% of total immune cells).

Stromal cells, like macrophages, were dramatically enriched in PNs (Figure 1C). Among the top 50 genes expressed in all fibroblast clusters was *Il33*, encoding a cytokine and transcriptional regulator often associated with inflammation. Nerve-resident endoneurial fibroblasts were relatively reduced. Perineurial cells, fibroblasts that form the blood-nerve barrier, did not change in abundance (c12; *Cav1*, *Ptch1*, *Slc2a1*, *Mpzl2*, *Lypd2*, and *Cldn1*) (33). Fibroblasts 1 (c0) was marked by *Cd34*, *Apod*, *Inhba*, *Dcn*, *Lum*, and the inflammatory cytokines *Cxcl1*, *Ccl11*, and *Ccl2*. Fibroblasts 2 expanded in tumors (c4; *Ptx3*). Cells in each fibroblast cluster (and SC clusters) upregulated transcripts that encode collagens (Supplemental Figure 3) and other matrix components, but we note that extended processing time can affect stromal cell gene expression (35). We also identified increased proportions of endothelial cells (c13; top unsupervised marker *Flt1*; the top 50 markers included *Pecam1*, *Cdh5*, *Egfl7*, *Emcm*, and *Esam*) (33). There was an unexpected increase in proportions of pericytes (c17; *Acta2*, *Myh11*, and *Myl9*) in PNs.

To define changes unique to tumors we assessed pairwise scRNA-seq gene expression changes between PNs and age-matched controls. Intriguingly, there were global changes in gene expression across all the identified clusters. Among the 21 cell populations with sufficient representation in both tumors and controls we identified 4,422 differentially expressed genes, associated with both commonly disrupted gene expression changes across cell populations and cell-type-specific differences. These genes include those broadly predicted to be upregulated by the transcription factors Jun, NF-κB1, and Yy1, and transcripts associated with basal lamina and β1 integrin cell surface interactions. Conversely, broadly downregulated genes were enriched in previously experimentally observed targets of Spi1, which is associated with transcripts mediating energy metabolism.

To verify that the identified populations and global changes represent populations detectable in unperturbed PNs, we compared the scRNA-seq expression data to bulk RNA-seq gene expression data from control DRGs/nerves (*n* = 3) and PNs (*n* = 11; *n* = 5 slow growing and *n* = 6 fast growing). This analysis found 890 transcripts deregulated in the same direction in single cells as compared to bulk profiles, indicating that bulk profiles are an agglomeration of diverse cell populations (immune, stromal, Schwann, neuronal) and broad *Nf1*-induced gene expression changes (Figure 1D). We hypothesized that loss of Nf1 in SCs causes transcriptomic changes not only in SCs, but also in immune and stromal cells, as robust changes are present in immune and stromal cells in tumors versus controls, and transcriptional reprogramming is demonstrable both by bulk RNA-seq and single-cell analysis.

### Mouse neurofibroma SC populations.

**Figure 2:**
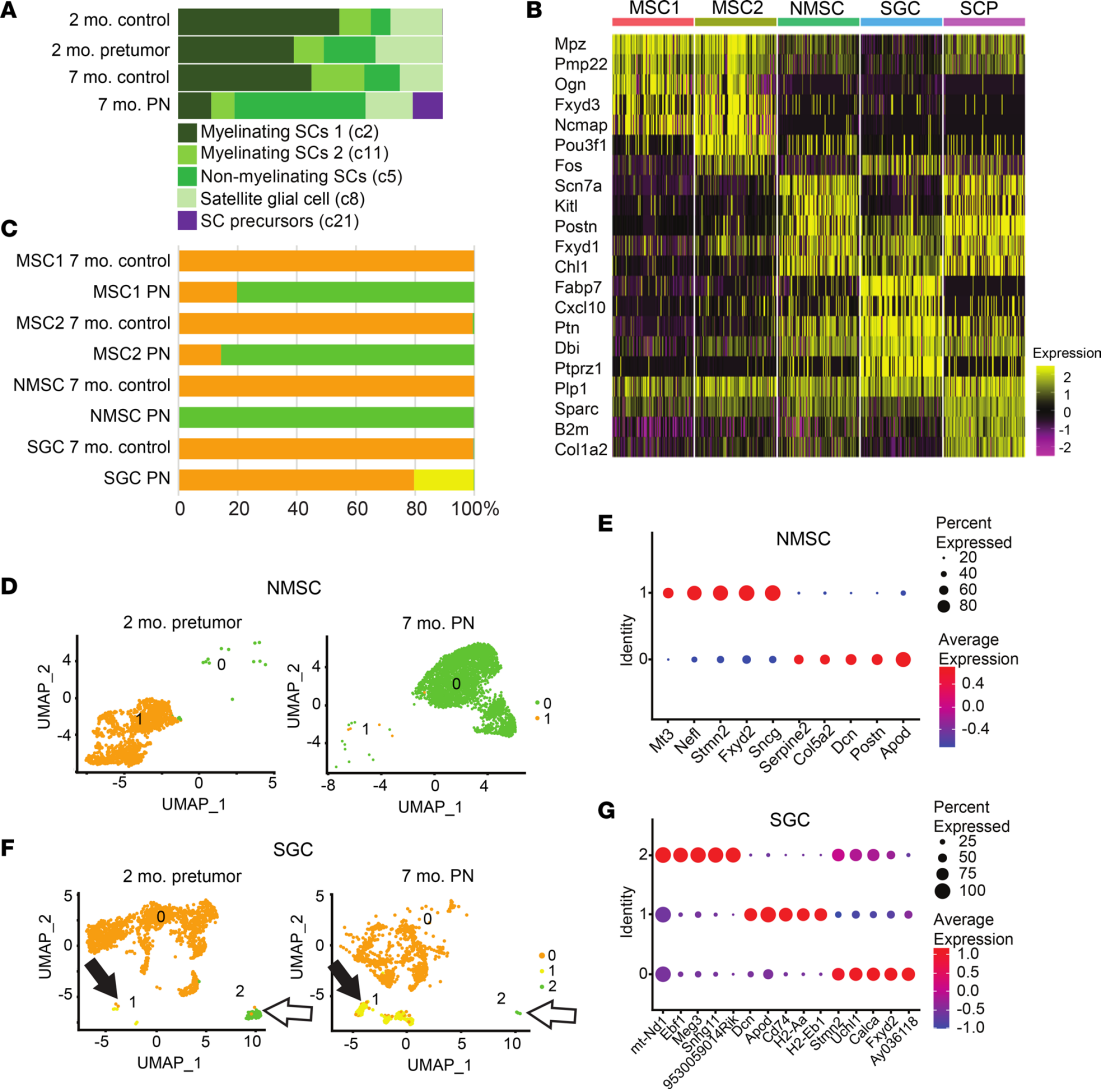
Normal SC clusters change abundance and gene expression in PNs, and PNs contain SCP-like cells. (**A**) Cell type frequencies across SC UMAP clusters. For each cell type, a UMAP cluster number (from **B**) is given in parentheses. (**B**) Heatmap showing the top 5 markers for each cluster, generated in Seurat. Shared markers are shown once. For visualization, each cluster was subsampled to 100 cells during heatmap generation. (**C**) Bar graph shows the percentage of SCs in each cluster that are normal (orange) or show changed gene expression in PNs (green or yellow). (**D**) A UMAP of NMSCs shows the shift in gene expression in PNs revealed by subcluster analysis. (**E**) Dot plot showing the top 5 markers for NMSC subclusters. On the *y* axis, “Identity” numbers represent cell subcluster numbers. (**F**) A UMAP of SGCs shows the shift in gene expression in PNs revealed by sucluster analysis; 1 SCG appears transiently (white arrows), the other (cluster 1) enlarges in PNs (black arrows). (**G**) Dot plot showing the top 5 markers for each of 3 SCG subclusters (green and yellow in **F**).

Given that SCs are the only cells with Nf1 loss in the *Dhh-Cre;Nf1^fl/fl^* model, we closely analyzed the 5 identified SC populations; the relative abundance of 3 of 5 SC populations changed on PN formation (Figure 2A). Dissociation methods and sorting methodology can limit SC population recovery. Recent quantification of SCs by nuclear single-nuclei RNA-seq (snRNA-seq) found that myelinating SCs (MSCs) accounted for 73.5% of all SCs, and MSCs plus NMSCs for 40% of the total cells in the sciatic nerve (36). Our dissociation and sorting of wild-type DRGs/nerves showed similar percentages; MSCs accounted for 25.4% and NMSCs for 4.8% of total cells, and of SCs, 70.4% were MSCs and 13.4% NMSCs. As expected, SGCs were also present in our proportions and accounted for 5.4% of total cells and 16.2% of SCs. Therefore, while dropout remains a potential concern, these results suggest that we captured representative cell populations.

Figure 2B shows a heatmap displaying expression of the top 5 gene markers that define each cell cluster. Cell-type-specific changes in gene regulation were associated with largely distinct gene regulatory programs, especially in MSC 2 and NMSCs. The top 50 markers/SC cluster (Supplemental Table 2) were used for comparison to available marker lists (10, 18, 27). Importantly, none of the PN SC populations, including NMSCs, showed expression of *Olig1*, *Shh*, or *Artn*, which mark the SCs lacking axonal contact after nerve injury (e.g., repair SCs; ref. 37); however, these genes are not highly abundant even after nerve injury, so some caution in interpretation may be warranted. Rather, 4 of 5 PN SC populations resembled recently described normal SC populations; the fifth resembled SCPs.

MSC 1 (c2) and MSC 2 (c11) were relatively enriched in characterized markers *Pmp22*, *Cryab*, *Mal*, *Mpz*, and *Cldn19*. Two MSC clusters were also identified by Gerber et al. (27). Importantly, *Pou3f1* (also known as *SCIP* or *Oct6*), normally a marker of immature/promyelinating SCs, was enriched in both of these PN clusters and was the in the top 2 markers of MSC 2. MSC 2 also expressed *Fos* and *FosB*, suggesting *Nf1* inactivation or Ras pathway activation (38). The expression of immediate early genes can result from long dissociation times, but our finding that only one of the MSC clusters expressed *Fos* and *FosB* suggests specificity unrelated to dissociation. The percentage of MSCs diminished as other SC types expanded in PNs.

In PNs, NMSCs (c5) are abnormal Remak cells associated with one or a few axons; NMSCs increased 3.1-fold in PNs (49% versus 13% in controls), consistent with NMSC proliferation in vivo (29). NMSCs were enriched in *Anxa1*, *Gfap*, and *Sox2*, as well as the NMSC-specific transcripts *Scn7a* and *Chl1*. NMSCs also showed enriched expression of the genes *Ptn*, *Ank3*, *Emp1*, and *Jun*, normally enriched in transition SCs, which are differentiating NMSCs (27). The SC genes *Foxd3* and *Gdnf* were also enriched in this PN cluster (39). PN SGC (c8) were defined by expression of *Fabp7* (40) and *Cxcl10*.

SCP-like cells (c21) accounted for 11% of the SCs in PNs. In contrast, only 2 cells were SCP-like in 2-month controls, none in 7-month controls, and 5 in 2-month *Dhh-Cre;Nf1^fl/fl^* pretumors. These cells therefore develop and/or expand as PNs form. SCP-like cells were enriched in transcripts characteristic of multiple stages of the SC lineage. The top gene in this cluster was *Postn*, which is normally an SCP/immature SC marker. *Tnc* is also an SCP/immature SC marker. *Chl1* and *C4b* normally mark NMSCs, *L1cam* marks transition SCs or NMSCs, *Sparc* and *Gatm* mark transition SCs, and *Serpine2* marks immature or promyelinating SCs (27, 41). *Ccnd1*, normally enriched in SCPs, immature SCs, or promyelinating SCs, is a marker of cell proliferation. *C4b* and *Sox4* were among the top 50 markers in PN NMSCs and SCPs. C4b is a complement pathway component normally expressed in NMSCs. *Sox4* marks SCs in peripheral neuropathy and is normally expressed in immature and promyelinating SCs (27, 41).

**Figure 3:**
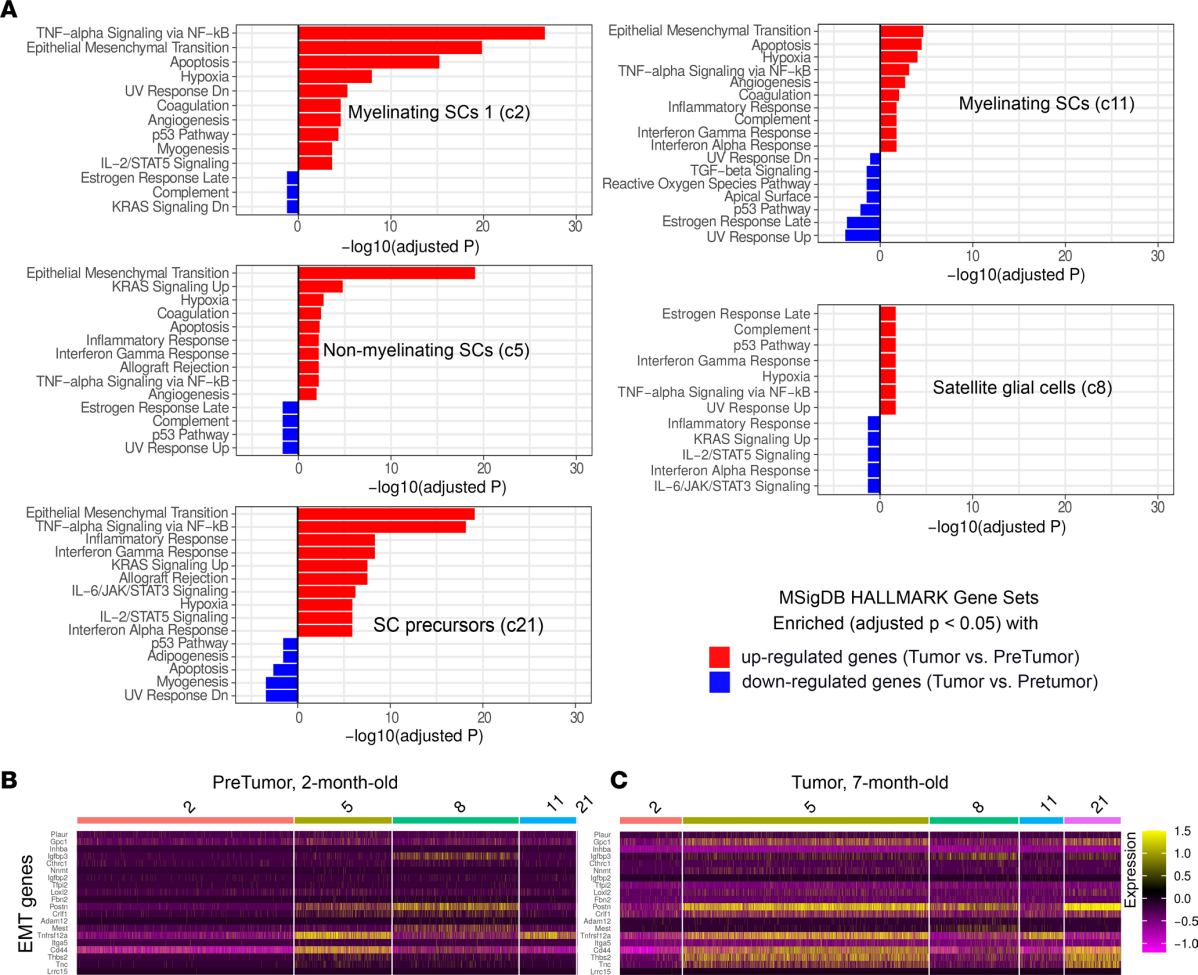
Enriched HALLMARK gene sets in 7-month-old tumor compared with 2-month-old pretumor SC clusters. (**A**) Enrichment analyses using up- and downregulated genes and MsigDB HALLMARK gene sets. (**B** and **C**) The EMT genes’ expression patterns in pretumor and tumor SC clusters. Differentially expressed genes were chosen by applying |fold change| > 1.5 and *P* < 0.05 filters.

Loss of *Nf1* in SCs occurs in the Dhh model at E12.5, yet few SC genes showed altered expression in 2-month-old *Dhh-Cre;Nf1^fl/fl^* cells (Supplemental Table 4). To define *Nf1-*driven changes, we compared each SC cluster in 7-month PN SCs to controls. Subclustering revealed changes in each SC population that were unique to PNs (Figure 2, C–G). Most PN SCs were also distinguishable from normal cells in uniform manifold approximation and projection (UMAP) plots; 100% of MSC 1, 80% of MSC 2, and 75% of NMSCs separated from normal cells. *Dcn*, *Apod*, and *Cd74* each showed increased expression in multiple SC clusters. Top upregulated genes per SC subcluster in PNs were MSC 1 (*Timp3*, *Sdc4*, *Dcn*, *H2-Ab1*, and *Cd74*), MSC 2 (*Apod*, *Dcn1*, *Cd74*, *Lum*, and *H2-Ab1*), NMSCs (*Apod*, *Postn*, *Dcn*, *Col5a2*, and *Serpine2*), and SGCs (*Dcn*, *Apod*, *Cd74*, *H2-Aa*, and *H2-Eb1*). The extent of gene expression change from 2 months to 7 months is shown for NMSCs (Figure 2, D and E). Only SGCs showed changes at 2 months; an SGC subcluster present at 2 months in only a few cells expanded by 7 months (Figure 2C). Pathway enrichment analysis of expanding SGC subcluster 1 using ToppGene (https://toppgene.cchmc.org) revealed enrichment of genes associated with mouse phenotype “Decreased response to injury” (Mouse Genome Informatics [http://www.informatics.jax.org/vocab/mp_ontology] mammalian phenotype term ID MP:0001876; *P* = 2.2 × 10^–11^). Pathway analysis showed “lung fibrosis” (WikiPathways [https://www.wikipathways.org] pathway ID M39477; *P* = 5.9 × 10^–15^). This SGC subcluster contained numerous cytokines and deserves further analysis. Based on these results, we performed enrichment analyses using Enrichr (https://maayanlab.cloud/Enrichr) by comparing MSigDB HALLMARK gene signatures between pretumors and tumors for each SC cluster (Figure 3A). MSCs in tumors (C2) showed response to injury reported for normal mice at early time points (39), while in those MSCs in C11 the responses were suppressed. NMSCs and SCP-like cells were notable for continued epithelial-mesenchymal transition (EMT) response in tumors; SGCs showed limited expression of injury response genes. After nerve injury, bridge cells (e.g., crush site cells through which axons will regenerate) and the distal nerve stump SCs alter gene expression and take on some mesenchymal traits, including an increase in EMT (39). We visualized SC-injury-associated EMT genes in pretumor and tumor SC clusters (Figure 3, B and C). Few showed significant expression, indicating that this response to injury in tumor SCs differs from that of nerve injury. Also, we note that in neurofibroma mouse models, tumors form around peripheral ganglia and in proximal peripheral nerve trunks. In contrast, nerve injury studies used mouse peripheral (sciatic) nerves and not ganglia. Therefore, RNA from different regions of the peripheral nervous system were used for these comparisons; further studies are required to determine whether regional variation in gene expression is a significant limitation of this study.

### NF-κB–induced pathways are activated in PN tumors.

**Figure 4:**
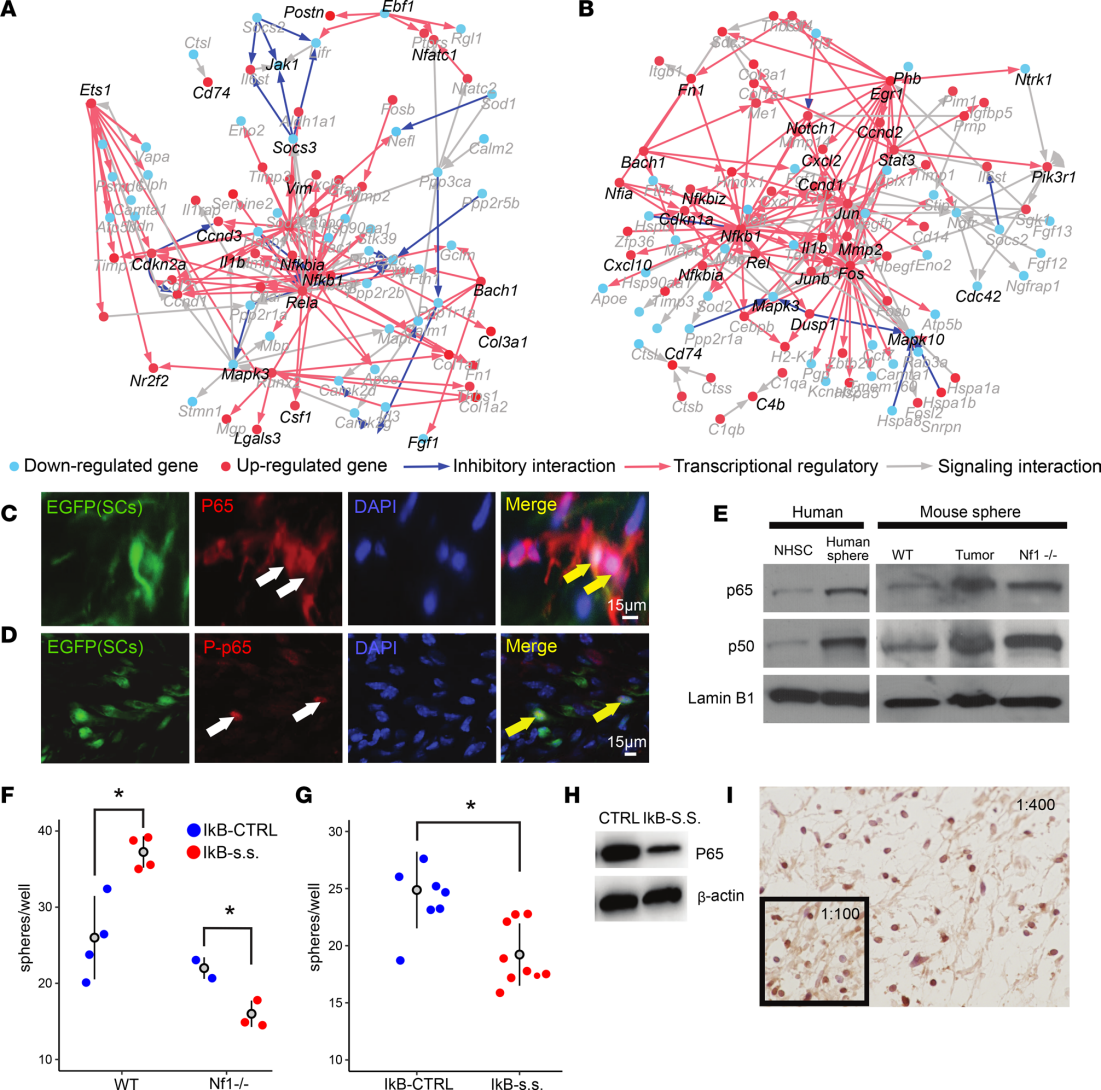
Network analysis predicting NF-κB–deregulated PNs confirmed by histological and in vitro analyses. (**A** and **B**) Differentially expressed gene network plots (cellHarmony) showing the central hubs in the NMSC PN cluster (**A**) and SGCs (**B**), containing NF-κB transcription factors. Genes shown adjacent to red dots are upregulated, and those next to blue dots are downregulated in 7-month PNs versus 7-month control. (**C** and **D**) Immunostaining of tissue sections shows the NF-κB protein p65 (red; **C**) in mouse PN SCs (expressing EGFP; green) and activated, phosphorylated, p65 (red; **D**) in mouse PN SC nuclei (green). Yellow arrows indicate colocalization. Scale bars: 15 μm. (**E**) Normal human SCs (NHSCs) express less p60 and p50 than sphere-forming cells from human PNs (human sphere). In mouse, SCPs from embryonic DRGs contain less p60 and p50 compared with either SCP-like cells from PNs or mouse *Nf1^–/–^* SCPs. Lamin B1 was used as a loading control. See complete unedited blots in the supplemental material. (**F** and **G**) Numbers of mouse *Nf1^–/–^* embryonic SCP spheres (**F**) and human SCP-like cells (**G**) are slightly reduced by infection with a dominant negative NF-κB (IκB-SS). *P < 0.05 by Welch’s *t* test. (**H**) Western blot confirming downregulation of p65 by IκB-SS. (**I**) p65 immunoreactivity in many cells in human PN tissue sections (1:400). Inset: At 1:100, 38% of cells show immunoreactivity.

To identify specific molecular programs induced in PN SCs, we performed network analysis of genes and predicted upstream regulators in cell populations identified in PNs using the software cellHarmony (42) (Figure 4). Both NMSCs and SGCs showed prominent regulatory and signaling interactions that highlighted NF-κB, RAS/MAPK, and STAT pathway activation; similar putative regulatory impact was also observed in other SC clusters (Supplemental Figure 4). In NMSCs this included upregulation of the RAS/MAPK target *Ets1* and downregulation of its targets (Figure 4A). Coordinate upregulation of NF-κB components (*Nfkbia*, *Nfkb1*, and *Rela*) and their targets (e.g., *Lgals3*, *Nr2f2*, *Csf1*, *Pdgfa*, and *Fgf1*) was observed in these cells along with factors mediating STAT-induced-feedback STAT inhibitor family (e.g., *Socs3*), indicating pathway activation.

In SGCs we identified upregulation of the RAS/MAPK pathway components *Fos*, *JunB*, *Jun*, and *Egr1*, and pathway target genes including *Dusp1*, a negative regulator of RAS/MAPK (Figure 4B). The NF-κB pathway was the central node in this cluster; *Nfia*, *Rel*, *Nfkbiz*, *Nfkbia*, and *Nfkb1* were upregulated, as were their downstream target genes *Cxcl10* and *Vim*; RAS/MAPK and NF-κB were predicted to converge to increase *Il1b* expression, confirming gene expression analysis in sorted PN SCs (43). *Stat3* was also upregulated; STAT3 is critical in PN SCs (17). Thus, after *Nf1* loss, transcriptional reprogramming in SCs, and activation of the NF-κB pathway, tumor formation/transcriptional reprogramming are observed in tumor immune cells and stroma. To test whether NF-κB is active in PNs we carried out double labeling. PN SCs expressed a GFP lineage tracer marking *Nf1* recombination. DAPI^+^ nuclei containing detectable, activated (nuclear) p65 represented 2.22% ± 0.33% of tumor cells, and, of GFP^+^ *Nf1*-mutant SCs, 7.12% ± 1.15% of mutant SCs do so. Thus, GFP^+^ cells were enriched for expression of nuclear, active, phosphorylated p65 protein, a readout of NF-κB pathway activity (Figure 4, C and D).

Ex vivo, SCP-like colonies from human PNs showed more p65 and p50 than normal human SCs on Western blots, and SCP-like colonies from mouse PNs showed elevated p65 and p50 as compared with SCP colonies from wild-type embryos; SCP colonies from *Nf1^–/–^* embryos were intermediate (Figure 4E). A specific dominant negative form of IKα, which irreversibly binds NF-κB (p65/p50) in the cytoplasm to inhibit p65 cytoplasm-nuclei translocation (44), slightly reduced numbers of sphere-forming cells from primary SCP spheres and significantly reduced *Nf1^–/–^* sphere number (Figure 4G) and resected primary human PN SCP-like spheres (Figure 4F). This correlated with decreased levels of p65 on Western blots (Figure 4H). Finally, 38.1% of tumor cell nuclei in human PN tissue sections showed nuclear anti-p65 immunoreactivity (Figure 4I), consistent with the idea that NF-κB–deregulated SCs affect the tumor environment. NF-κB and STAT3 signaling pathways in SCPs and SCs likely contribute to expression of cytokines, chemokines, and growth factors to maintain ongoing inflammation and recruitment of immune cells in PNs, and activation of stromal cells.

### Broad cellular impacts in mouse NF are reproduced in human NF.

**Figure 5:**
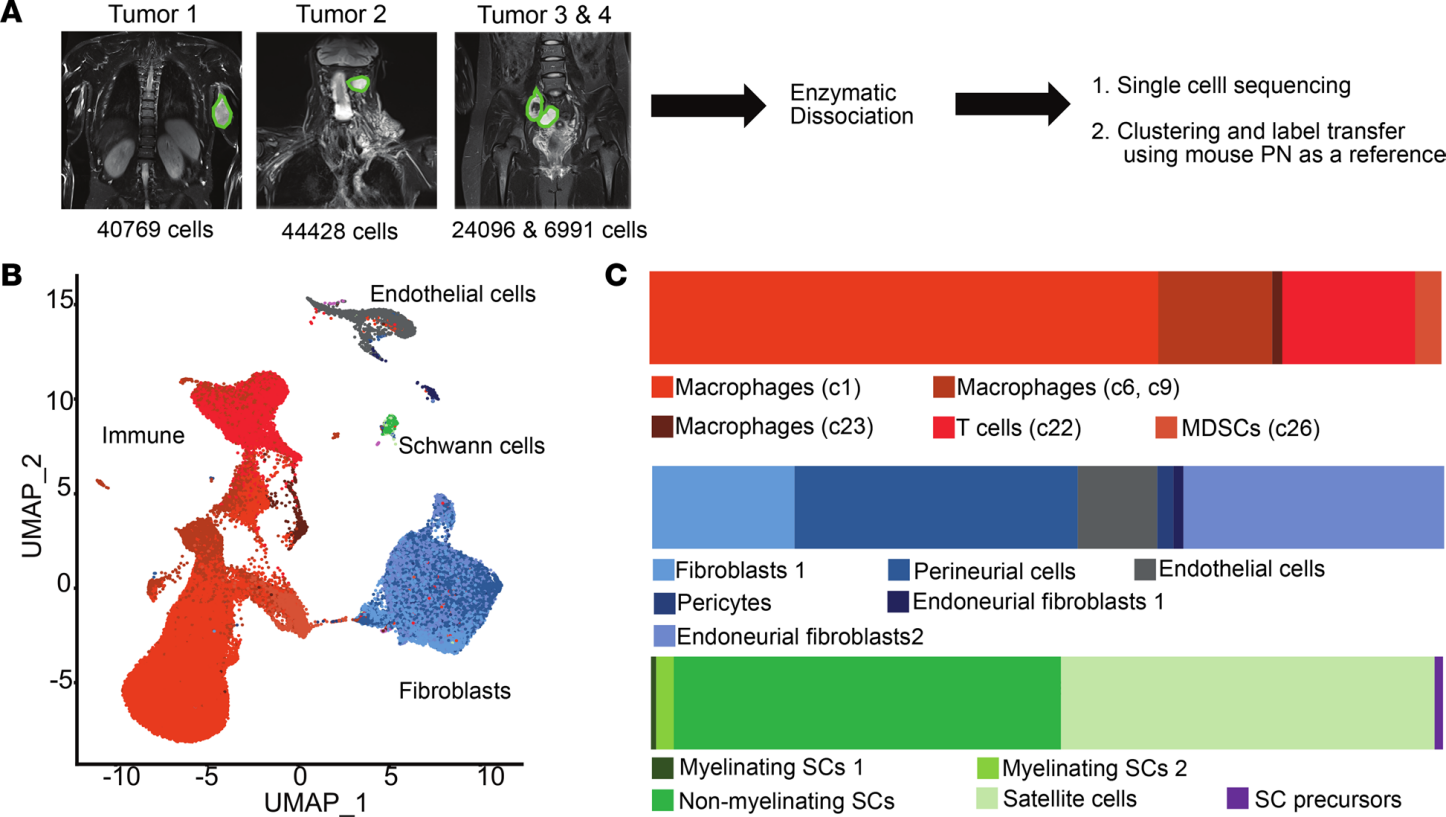
Shared cell types between GEMM and human PNs. (**A**) Schematic of experimental design for single-cell profiling of human PNs and label transfer to enable comparison to mouse PNs. (**B**) UMAP shows results of human PN analysis, with annotations after mouse label transfer (Seurat). Note the relative paucity of SCs. (**C**) Cell type frequencies across sample types in **B** show multiple types of immune (orange) and stromal (blue/gray) cells. SCs (green) and SCP-like cells (purple) are detectable.

Thus, after *Nf1* loss, transcriptional reprogramming in SCs and activation of the NF-κB pathway and tumor formation/transcriptional reprogramming in tumor immune cells and stroma are observed. To identify the transcriptional changes in PN GEMM that are shared in human PNs, and therefore potentially targetable, we compared human and mouse PNs. Whole-body imaging identified 4 PNs in 3 NF1 patients (Figure 5A), which were resected for clinical indications. The lesions were anatomically located in the left upper extremity associated with the median nerve (tumor 1), the extradural region of the posterior left cervical spine (tumor 2), the superficial inguinal region (tumor 3), and the pelvis associated with the lumbar plexus (tumor 4). MRI and operative findings demonstrated that these lesions were located distal to the DRGs, where neuronal cell bodies and their attendant SGCs are located. The tumors were dissociated and profiled using scRNA-seq; similar processing time and methodology were used in mice and to minimize differences between species. We also used the same analytical pipeline to process the human data. In human PNs, 30 unique clusters were identified (Supplemental Figure 5). To assess the reproducibility of observed PN cell populations from mouse to human, we projected the mouse PN cell populations to all human PN cells. This analysis found that of the 31 mouse clusters, 20 clusters were readily identified in human PNs; the majority were not observed in normal mouse controls (Figure 5B). No neurons (which accounted for 8 mouse clusters) were identified in human PNs, attributable to surgical avoidance of peripheral ganglia during human tumor resection. Notably, all 5 SC populations were detected in human PN samples, including SCP-like cells and cells expressing markers of SGCs in human PNs (*FABP7* and *CXCL10*). NMSCs showed strikingly similar proportions in mouse and human, at 49% of total SCs (Figure 5C).

Notable differences between mouse and human NF were that human PNs contained smaller proportions of SCs and far greater proportions of immune and stromal cells (Figure 5C). For example, only 0.7% of human PN cells were SCs, as compared with 14% in mouse PNs. Progressive changes in human tumors likely explain this result, although there are known immune cell differences between species. Human PNs had no Fibroblast 2 cluster cells, which accounted for 26% of mouse PN stromal cells.

### Receptor-ligand interactions in PN.

To identify potentially actionable cellular interactions that underly the substantial inflammatory response effects in PN, we searched for altered receptor-ligand interactions using the software CellPhoneDB (45). Although such analyses are typically hindered by an excess of false positive predictions, we were able to leverage conserved receptor-ligand interactions, observed in the same cell populations in both mouse and human PNs, and absent in controls (Supplemental Table 5). From this analysis, we found that the MSC 1 cluster is not predicted to have frequent receptor-ligand interactions that are unique to tumors (22 interactions), while the MSC 2 cluster is predicted to have some (141 interactions), and NMSCs and SCP-like cells many (286 and 216 interactions, respectively). We analyzed those genes with predicted altered expression in SC clusters, after manual annotation to verify ligand/receptor status in the literature and evaluating expression levels for elevated expression in mouse tumors (not shown).

**Figure 6:**
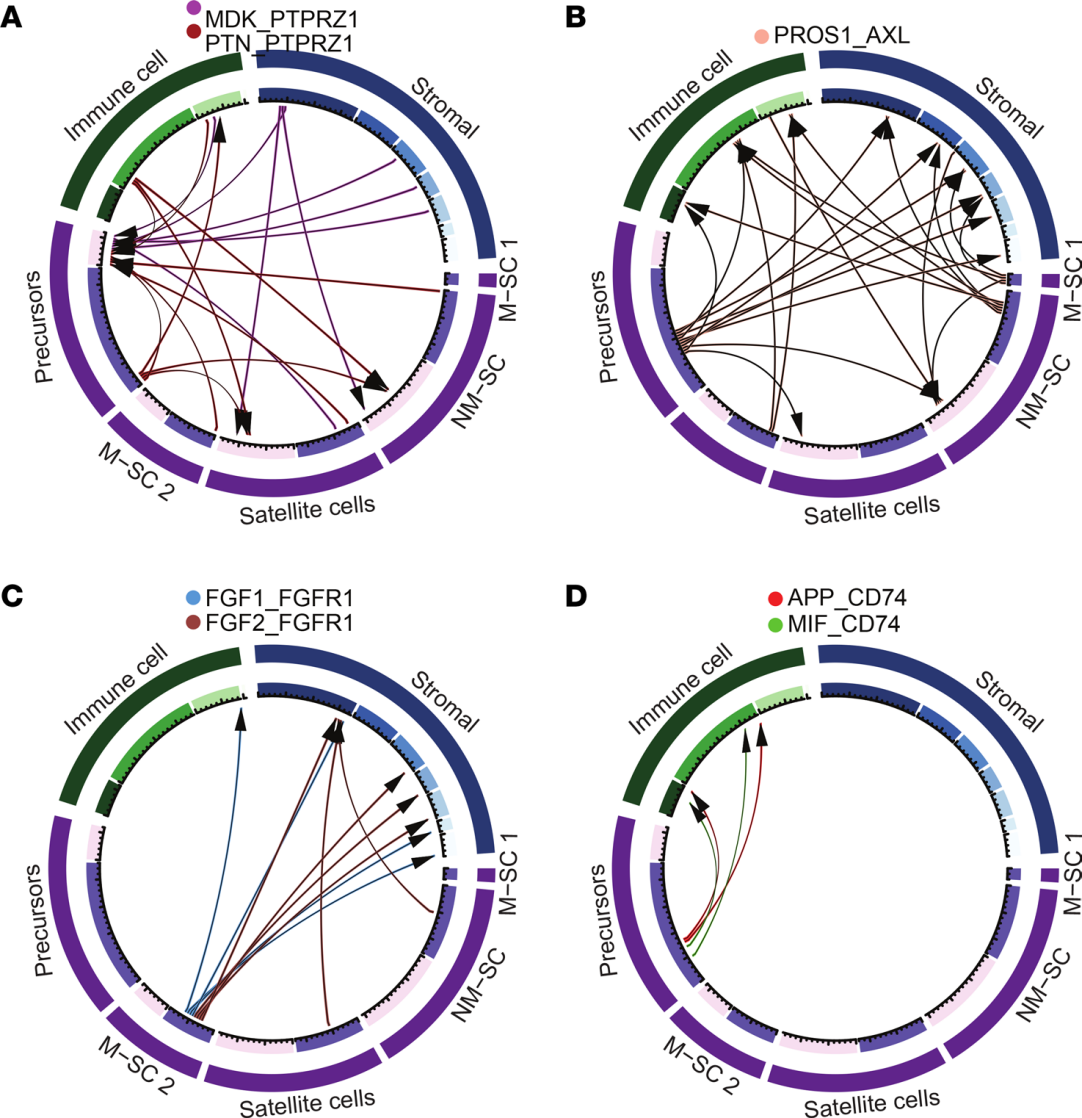
CellPhoneDB analysis of predicted major SC-cell interactions in mouse and human PNs. CIRCOS plots. (**A**) Immune cells, stromal cells, and SCs produce midkine (MDK) and pleiotrophin (PTN); these ligands act mainly via the phosphatase PTPRZ1 in SCP-like cells. (**B**) SCP-like cells and myelinating SCs are predicted to produce PROS1, a ligand for the AXL receptor; AXL is present on immune cells, stromal cells, and some SCs. (**C**) Myelinating SCs are predicted to produce FGF1 and FGF2, affecting largely stromal cells through the receptor FGFR1. Other SC types are also predicted to express FGF1. (**D**) SCP-like cells are predicted to produce the ligands APP and MIF, affecting immune cells via the CD74 receptor. M-SC, myelinating SC; NM-SC, nonmyelinating SC.

We found that SC types upregulated expression of ligands predicted to influence other SCs and/or stromal/immune populations (Figure 6, A–D). For example, PTN (pleiotrophin) and MDK (midkine) are homologous ligands known to increase in response to inflammation and reported to influence many aspects of cancer biology, which are transcriptionally responsive to NF-κB. Both ligands bind the tyrosine phosphatase PTPRZ1 and other cell surface receptors (46). *MDK* and *PTN* transcripts were elevated in mouse *Nf1^–/–^* SCs, and MDK immunoreactivity was detected in PN SCs (47). Also, PROS1 was expressed by stromal cells, immune cells, and some SCs. Via the AXL receptor, PROS1 is predicted to affect SCP-like cells, other types of SCs, and stromal/immune cells (Figure 6B). *FGF1* and *FGF2* were predicted to be mainly produced by MSC 2, and to influence receptors on stromal cell populations (Figure 6C). Thus, different sets of ligands are produced by specific types of tumor SCs, and affect various types of SCs, immune cells, and stroma. In addition, human and mouse SCP-like cells and NMSCs expressed *C4b* (complement factor 4b), which is necessary for production of downstream complement peptides that modulate innate immune responses. The complement receptors *C5ar1* and *C3ar1* were predicted to be expressed on some PN macrophages, DCs, and other immune cells.

**Figure 7:**
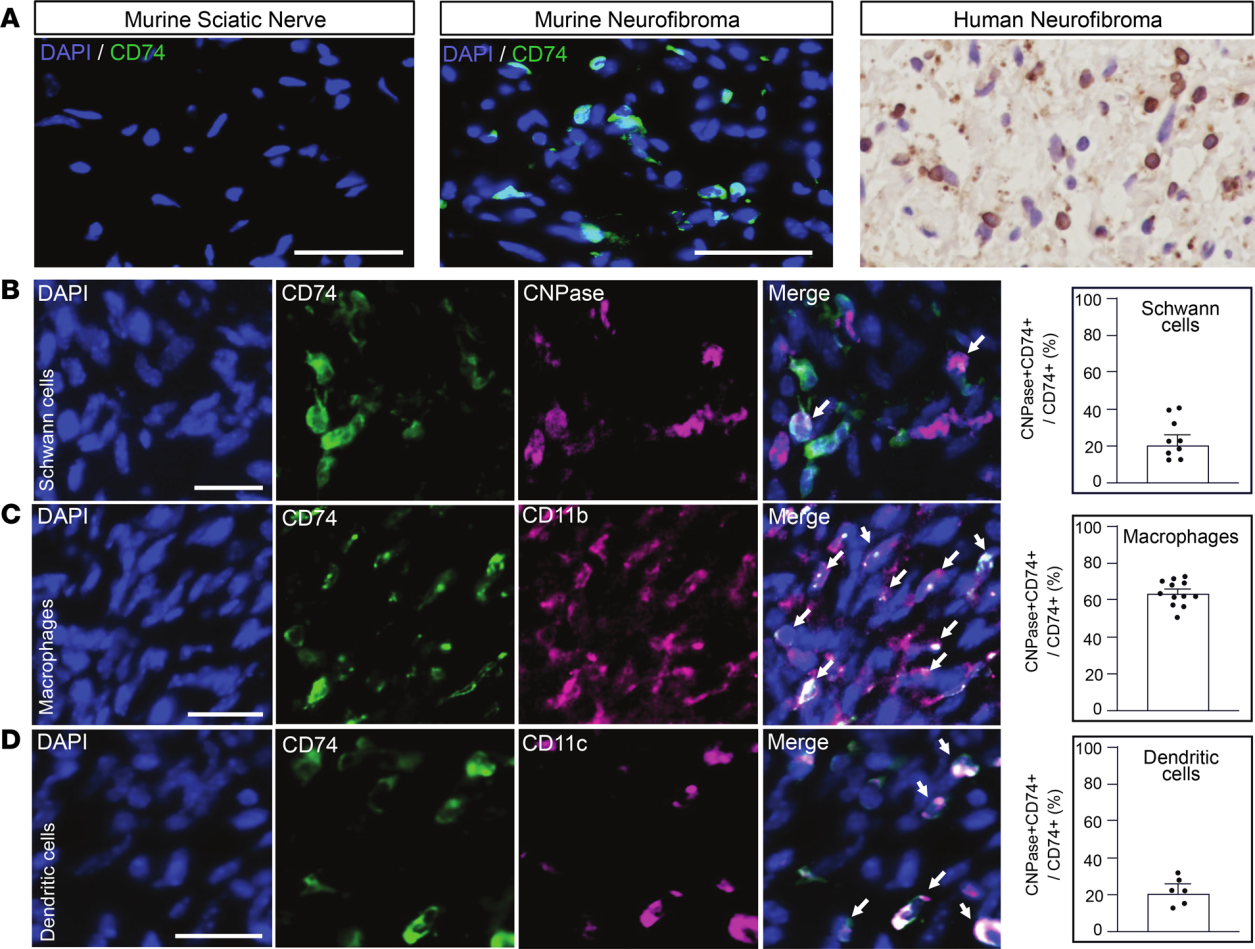
CD74 is expressed by PN SCs, macrophages, and DCs. (**A**) Immunostaining reveals that CD74 expression (green) is low or absent in normal mouse sciatic nerve; expression is elevated in some cells in mouse PNs. Nuclei are stained with DAPI (blue). At right, in human PN tissue sections, CD74 (brown) is expressed by many cells; counterstain is purple. (**B**) CD74 colocalizes with SCs (CNPase, **B**), macrophages (CD11b, **C**), and DCs (CD11c, **D**) in mouse PNs (white arrows denote colabeled cells). Scale bars: 50 μm. In **B**–**D**, dot plots quantify the percentage of cells stained/PN. Horizontal bars denote mean ± SEM.

SCP-like cells, in mouse and human, uniquely expressed the ligands macrophage migration inhibitory factor (*MIF*) and *APP* (Figure 6D). Expression of *CD74*, the MIF receptor, was predicted to be enriched in immune cells, especially macrophages/DCs, and to a lesser extent in SCPs (data not shown). CD74 immunoreactivity was undetectable in normal mouse nerve, and dramatically increased in mouse PNs (Figure 7A). Most human PN cells showed CD74 staining (Figure 7A). Double labeling CD74^+^ cells with antibodies recognizing CNPase (SCs; Figure 7B), CD11b (macrophages; Figure 7C), and CD11c (DCs; Figure 7D) revealed that of CD74^+^ cells, an average of 20% were CNPase^+^ SCs, 60% were CD11b^+^ macrophages, and 20% were CD11c^+^ DCs (Figure 7, B–D), validating our identification of factors upregulated in PN. The receptor CD74 can activate NF-κB and RAS/MAPK pathways and is potentially targetable in each of these cell types.

**Figure 8:**
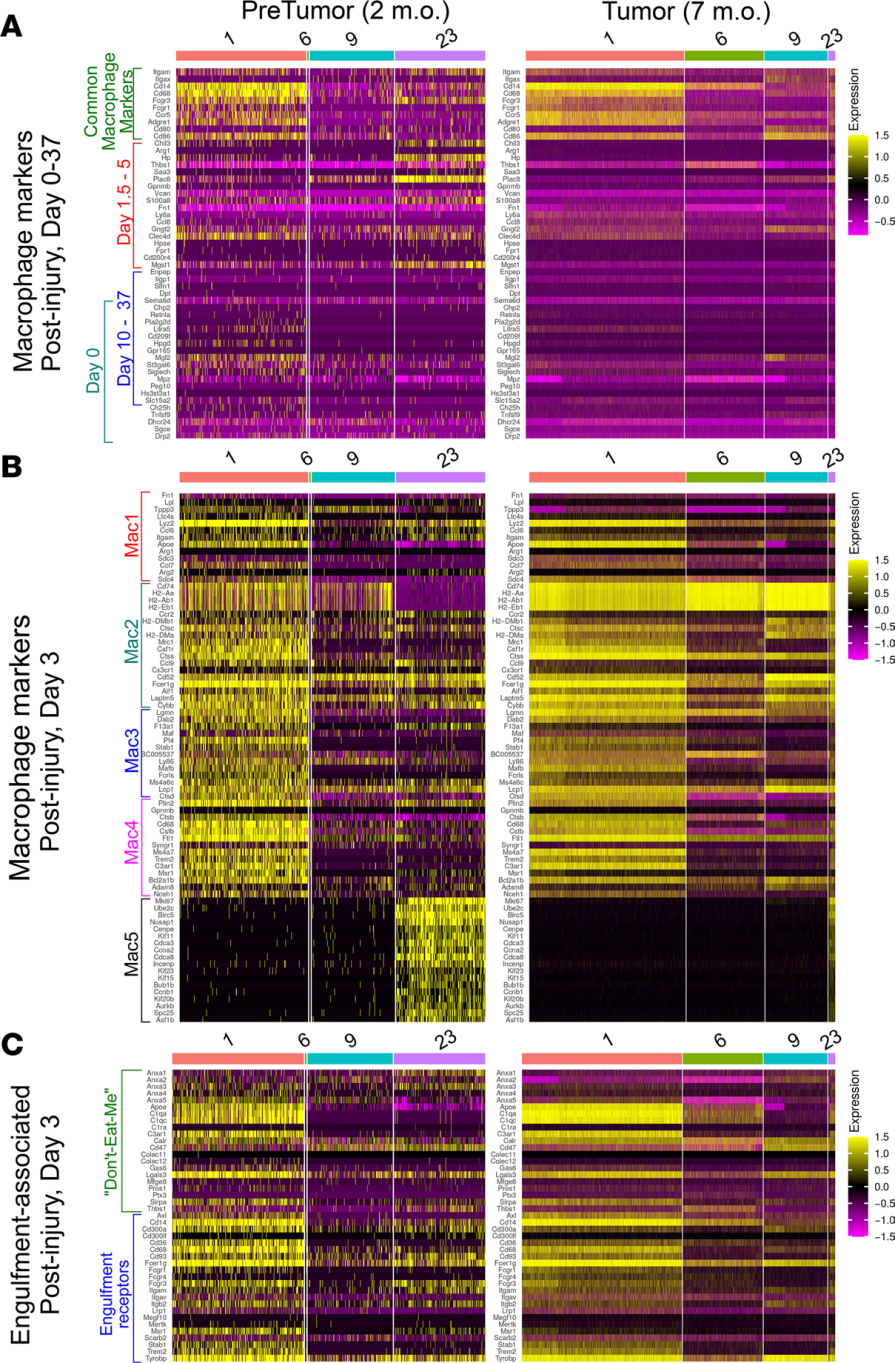
The gene expression pattens of macrophage clusters. Based on (**A**) days after nerve injury (48), (**B**) nerve injury-associated macrophage subtypes (49), and (**C**) “don’t-eat-me” and “eat-me” (engulfment receptor) signatures (49).

Upregulated expression of cytokine/chemokine/growth transcripts that we observe in PNs was also described after nerve injury. After nerve crush, there is a robust influx of immune cells into nerves. We compared PN macrophage subsets to the macrophage populations defined after nerve injury, annotating clusters based on literature review and analysis of developing, adult, and injured murine peripheral nerve (33, 48) (Supplemental Table 3 and Figure 8). On days 1.5–5 after injury, macrophages were proinflammatory; by days 10–37 they resembled antiinflammatory macrophages in normal nerve (48). After normalizing expression across groups to enable comparisons across data sets, in pretumors (2-month *DhhCre;Nf1^fl/fl^*) only a few genes described as part of an early proinflammatory signature (*Saa3*, *Plac8*, *Clec4*, and *Mgst1*) were expressed (Figure 8A). In tumors, overall, genes characteristic of the proinflammatory response showed reduced expression (*Saa3*, *Plac8*, *Fn1*, and *Thbsp1*); only 2 of these proinflammatory genes remained increased (*Ly6a* and *Ccl8*). Thus, PN macrophages, like PN SCs, largely did not show signatures characteristic of the injury response at these time points.

To confirm this conclusion, we analyzed macrophage populations identified on day 3 after nerve injury (49), when expansion of the myeloid compartment peaks. We compared the top 20 genes from each of 5 macrophage clusters to pretumor and tumor macrophages. Cells in our c23 resembled “Mac5” cells, annotated as blood-derived, proliferating myeloid cells with stem-like features (e.g., monocytes), are prominent in protumors but rare in tumors. Expression of most Mac1–Mac4 genes (cells differentiating into nerve-specific macrophage types) was also decreased in tumors (Figure 8B). Exceptions to the notable decrease in injury-related genes is the elevated expression of MHCII genes (*H2-Aa*, *H2-Ab1*, and *H2-Eb1*) and the invariant chain of MHCII (*Cd74*), which are important for antigen presentation to T cells; expression of these genes is a characteristic of nerve macrophages. Another feature of nerve macrophages after injury is elevation of expression of phagocytosis-related “eat me” and “don’t eat me” genes (Figure 8C). Many of these genes, present in the Mac4 cluster, as in the setting of nerve injury, were expressed in pretumor macrophages. Like expression of most other injury-related markers, we observed blunted expression of engulfment-related genes in tumor macrophages.

## Discussion

We identified 5 types of SCs in mouse and human PNs, each with unique patterns of gene expression, including unique patterns of predicted cell-cell communication. Most PN SCs resemble normal SC populations: NMSCs, SGCs, and 2 populations of MSCs. However, each PN SC population developed patterns of gene expression that were different from those in normal nerve, including SC *NFkB*/*Stat3*/*Ap1* activation that correlated with the increased presence of immune and stromal cells in PNs. We identified SCP-like cells in PNs that were absent in controls. SCP-like cells upregulated expression of *MIF*, encoding a ligand for the receptor CD74 that can activate the NF-κB and Erk1/2 pathways (50, 51); *CD74* is expressed in PN SCs, macrophages, and DCs.

SC reprogramming was negligible at 2 months of age in most SC populations, more than 2 months after the genetic loss of *Nf1* occurs in these cells. *NF1*-mutant SCs therefore gradually rewire the PN microenvironment, to evade immune suppression and generate a permissive environment for tumor growth. PN NMSCs also differ from normal NMSCs by gradually elevating expression of markers of more immature SCs, those developing into mature Remak cells (27). The timing of altered NMSC gene expression correlates with the progressive Remak bundle disruption observed in PN models. Some of these SGCs (14, 29) are the first SC type that shows significant changes in PN gene expression. Two aberrant subsets of SGCs appear at 2 months of age, and one expands in PNs. Intriguingly, SGC-marker-containing cells were also detected in human PNs, although no neurons were present in the resected samples. We postulate that after loss of NF1, SC population(s) take on characteristics of SGCs. Our data are consistent with the observation that *Cxcl10* and *FABP* expression increases between 1 and 2 months of age in *Dhh-Cre;Nf1^fl/fl^* sciatic nerves; Fabp7 is normally a marker of normal SCPs and of SGCs in adult nerve (14).

Although normal adult nerves/DRGs do not contain stem cells, we identified an SCP-like population in PNs (20). SCP-like cells were present at low abundance (or absent) in 2-month *Dhh-Cre;Nf1^fl/fl^* samples. They expressed markers of SCPs, immature, transitional, and premyelinating SCs but did not express neural crest cell markers. *Ccnd1* was a unique top marker for the SCP-like population; *Ccnd1* is characteristic of proliferating progenitor cells, not stem cells. We posit that SCP-like cells arise through dedifferentiation of mature *Nf1^–/–^* SCs, as suggested in prior studies (29, 52). SCP-like cells increase in number in PNs; taken together with findings that *Nf1^–/–^* embryonic SCs show limited in vitro self-renewal capacity and form neurofibroma-like lesions in mice (24, 25), we conclude that these are progenitor-like cells with roles in PN formation and/or growth.

SCP-like cells are also marked by elevated expression of periostin (*Postn*), which is also upregulated by neurofibroma NMSCs. Periostin is an extracellular ligand for integrins; it also promotes fibrosis and is often expressed at sites of injury/repair and inflammation. In a model of polyneuropathy, and in injured nerve, periostin expression was also upregulated in SCs (39, 52). *Postn* deletion in cardiac nerve Remak cells resulted in nerve defasciculation (53). *Postn* deficiency also delayed and reduced neuropathy, and decreased numbers of nerve macrophages (54). *Postn* might play similar key roles in PN fibrosis and/or macrophage recruitment. Another gene with aberrant upregulated expression in PN MSCs and SGCs is *Dcn*, which encodes a secreted extracellular proteoglycan. Analyzing the contributions of these genes to PN should be a fruitful area for further study.

SC reprogramming occurs after *NF1* loss and is concomitant with NF-κB activation signatures in PNs. Reprogramming is unlikely to be genetic, as recurrent genetic lesions apart from those at *NF1* itself have not been identified in PN SCs (25, 55). When tumorigenesis is driven by mutant RAS, oncogenic stress and apoptosis initially occur (56, 57). This effect can be counteracted by activation of prosurvival pathways downstream of RAS, and by constitutive NF-κB activity, promoting tumorigenesis (57, 58). Factors released from tumor cells feed forward to increase activation of NF-κB (NF-κB1), STAT3, and AP1 (JUN, JUNB, and FOS) in surrounding cells (59, 60). Loss-of-function mutations in *NF1* activate RAS and are therefore likely to mimic RAS-mutation-mediated early stress/apoptosis.

Our scRNA-seq and bulk RNA-seq data analysis highlighted *NFkb*/*Stat3*/*Ap1* transcription factor response as deregulated in multiple PN SC subtypes, and deregulated in most immune and stromal cell types (including macrophages, endothelial cells, neurons, and fibroblasts). This analysis acts as a control for artifacts caused by tissue dissociation, which occurs over time and therefore can affect gene expression. *Nfkb* was also predicted to be a central node in SC gene regulatory network predictions. RAS activation drives Ap1 factor transcriptional activity. The relevance of STAT3 is consistent with genetic deletion of *Stat3* in PN SCs dramatically reducing PN initiation, growth, and PN macrophage number (15, 17). We confirmed NF-κB expression in PN SCs and immune cells and propose that blockade of NF-κB activity may also limit PN growth. Nonspecific NF-κB blockers such as salicylates, proteasome inhibitors, and glucocorticoids achieve partial inhibition of NF-κB (61). STAT3-, NF-κB–, and AP-1–regulated genes may also have combinatorial roles (60) in driving PN.

The production of cytokines/chemokines/growth factors is an important tumor-promoting mechanism, predicted to influence surrounding SCs, immune cells, and stromal cells. In normal nerve, MSCs express few transcripts encoding these factors (33) and a subset of PN MSCs aberrantly upregulate cytokine/chemokine/growth factor transcripts. Our results are consistent with identification of factors in human and mouse *NF1^–/–^* SC–conditioned medium that augment SC migration, immune cell migration, and angiogenesis (10, 43, 62–65). Some of the upregulated transcripts identified in this study have been studied in mouse or human NF1 SCs. Midkine is upregulated in SCs after injury, secreted by NF1 SCs, and is a known NF-κB target gene (47, 66). *FGF1* and *FGF2* were upregulated largely in MSCs in PNs. FGF1 is absent in normal SCs (67). FGF2 can be purified from human PNs (68) and is expressed by normal and injured SCs (69–71).

SCP-like cells express PROS1 (protein S), a ligand for phagocytic tumor-associated macrophage, (TAM) receptors, including Axl; the soluble form of AXL was proposed as a secreted biomarker for NF1 nerve tumors (72). PROS1 binds to phosphatidylserine (PtdSer) exposed on dying cells and upon binding TAM-family receptors induces efferocytosis; after injury, SCs express TAM receptors (73). SCP-like cells also express *MIF*. MIF/CD74 signaling can activate NF-κB and MAPK (50, 51, 74), so this pathway may reinforce effects of SCs on surrounding immune cells. CD74 contributes to tissue inflammation, tumor progression, and metastasis (50, 75, 76), and most evidence supports a therapeutic role for blocking CD74 in cancer. De Azevedo et al. (77) found that blocking the MIF-CD74 axis in melanoma models increased CD8^+^ T cell infiltration, promoted macrophage conversion to an M1-like phenotype, and showed a combination effect with anti-CTLA4. The approval of milatuzumab, a humanized CD74-targeting antibody in human multiple myeloma and non-Hodgkin lymphoma (78), and ongoing development of antibodies that target MIF (79) provide candidates to test for therapeutic effects on PNs.

In sum, scRNA-seq and cross-species analysis allowed insight into the striking diversity of SC populations in PNs. The data support the hypothesis that the SCP-like cells in PNs develop from mature SCs. We identify a rich tumor microenvironment composed of stromal and immune cells, including cell types that have not been studied in PNs to the best of our knowledge: MDSCs, several types of macrophages, and multiple types of fibroblasts, each of which may play roles in suppressing antitumor immunity and/or facilitating tumor growth. The studies demonstrate that the recruitment of multiple types of stromal and immune cells is a secondary effect of loss of *NF1* in SCs and reveal potential therapeutic targets, including NF-κB and CD74.

## Methods

### Sample acquisition.

Adult male and female *Dhh-Cre;Nf1^fl/fl^* mice on a largely C57BL/6 background were used (11). Fresh human PNs from debulking surgeries (*n* = 4 individual tumors) were taken directly from the operating room.

### Sample preparation.

Immediately following euthanasia, paraspinal tumors (2- or 7-month *Dhh-Cre;Nf1^fl/fl^*; *n* = 6), and age-matched wild-type DRGs with nerve roots (*n* = 2) were excised within 5 minutes and placed into ice-cold L-15 medium (Thermo Fisher Scientific, MT-10-045-CV). Tissue was cut into 1-mm^3^ pieces and placed into dissociation medium in 50 mL tubes containing 20 mL L-15 medium, 10 mg collagenase type I (Worthington Biochemical, LS004196), and 50 mg Dispase II (Sigma-Aldrich, 04942078001) in a 37°C incubator for 2 hours, with 170 rpm shaking. The dissociation process was stopped by adding 30 mL DMEM (Thermo Fisher Scientific, 11-965-1188) containing 10% FBS. Samples were centrifuged at 800*g* for 5 minutes at 4°C. Supernatants were removed and cell pellets were resuspended into 50 mL of 1× PBS containing 0.1% BSA. Suspensions were sequentially filtered with 70 μm (Thermo Fisher Scientific, 08-771-2), 40 μm (Thomas Scientific, 1181X52), and 20 μm cell strainers (pluriSelect, 43-50020-03) and centrifuged at 600*g* for 5 minutes at 4°C. Pellets were resuspended into 100 μL of 1× PBS/0.1% BSA. Trypan blue–negative viable cells were counted to determine the cell concentration for droplet-based scRNA-seq.

Procedures for human NF cells were the same as for mouse with the following modifications. Dissociation medium also contained DNase I (Sigma-Aldrich), and each tumor was dissociated on the gentleMACS dissociator (Miltenyi Biotec) followed with shaking in 37°C at 150 rpm for 45 minutes and 2 more rounds on the gentleMACS dissociator. The dissociated tumor cells were passed through a 40 μm cell strainer to achieve single-cell suspensions before counted using the Cellometer Auto 2000 cell viability counter (Nexcelom) with the presence of acridine orange and propidium iodide dyes.

### Library preparation.

Library synthesis was performed according to manufacturer’s instructions to generate barcoded cDNA libraries for scRNA-seq. Ten thousand viable cells per lane from each sample (mouse or human) were loaded using the 10× Chromium platform version 2 or 3 chemistries (3′) (10× Genomics). For mouse, 1 lane was loaded per sample, yielding a median of 8,416 captured cells per sample. In total, 80,968 mouse cells were included in this analysis. Overall sequencing depth per capture was approximately 250 million reads. Details for each mouse sample including reads per cell (which varied from 15,488–135,606) are shown in Supplemental Table 1. We performed 1 full-lane sequence on 2 paired-end 75-bp flow cells using an Illumina HiSeq 2500. For the human samples, 3 to 8 lanes of cells were loaded per tumor, yielding 4,589–40,871 cells for sequencing on an Illumina NextSeq sequencer. In total, 103,446 human cells were included in this analysis.

### scRNA-seq analysis.

The 10× scRNA-Seq libraries from mouse samples were aligned to the mm10 mouse genome using the Cell Ranger pipeline (version 3.0.2, 10× Genomics) with default parameters. Detailed Cell Ranger quality metrics for each sample are shown in Supplemental Table 1. Raw data, including FASTQs and count matrices, are available in the NCBI Gene Expression Omnibus database (GEO GSE181985). Downstream analyses were performed using Seurat (version 3.1.2) (80), Scrublet (version 0.2.1) (32), cellHarmony (AltAnalyze version 2.1.4) (42), and CellPhoneDB (version 2.1.2) (45). Seurat was used for data normalization, dimensionality reduction, clustering, and integration using the standard Seurat v3 integration workflow as described in Stuart et al. (80). For clustering functions, dims was set to 1:30 and resolution to 0.5. For discovery of SC subpopulations, resolution was set to 0.025 (clusters 2, 5, and 8) or 0.050 (cluster 11) to yield at least 2 subpopulations per cluster. During sample integration, default parameters were used in Seurat’s FindIntegrationAnchors function, including setting anchor features to 2000. Cell type cluster annotation was performed by comparing the top 50 cluster markers identified with the FindAllMarkers function in Seurat to published literature. The top 50 markers for each cluster are available in Supplemental Table 2 and visualized in Supplemental Figure 1. When calculating cell type proportions across different conditions, cell counts for clusters with fewer than 3 unique markers were merged with the most transcriptionally similar cluster. Other downstream analyses and visualizations did not merge cluster data unless otherwise noted. Scrublet was used for doublet detection. A threshold of 0.35 was manually set based on an examination of the simulated doublet scores distribution. Seurat numerical cluster annotation, cell type annotation, cluster merging, and calculated doublet percentages are shown in Supplemental Table 3. UMAP representation of doublet scores is shown in Supplemental Figure 2. cellHarmony was used to find network-level differences between clusters across different experimental groups (fold change > 1.2, empirical Bayes *t* test *P* < 0.05, FDR). Cluster assignment for cellHarmony analyses were based on Seurat output. CellPhoneDB was used to predict cell-cell interactions between clusters that are shared between human and mouse PNs. The default CellPhoneDB receptor/ligand database was used for this analysis. Example scripts with documentation of analysis parameters, cell barcode to cluster assignments, cell barcode to group assignments, and UMAP coordinates are available on Github: https://github.com/LKershner/neurofibroma Human scRNA-seq analyses were performed using the same above protocol (Seurat 3) with count matrices deposited in NCBI’s GEO (GSE181985).

### Bulk RNA-seq analysis.

We collected control DRGs/nerves and PNs from 9-month-old mice. We defined rapidly growing PNs as those with an increase in volume of greater than 35 mm^3^, and slowly growing PNs as those with an altered volume of less than 5 mm^3^, in a 2-month period, based on volumetric MRI scans performed at 7 and 9 months of age. We extracted RNA from frozen PNs to generate 35–40 million reads per PN. Total RNAs were isolated by phenol/chloroform methods and purified (Qiagen Mini RNA Isolation Kit, 74104). RNA libraries were amplified with the Ovation RNA-Seq System v2 (Nugen). Base calling was preformed using Illumina CASAVA (v1.4) and read quality checked with FASTQC. Reads were aligned against the mm10 mouse genome with TopHat. Raw gene counts were calculated using Subread/featureCounts (v1.5.2) and normalized using edgeR’s TMM method. Raw and processed sequencing data files are deposited in NCBI’s GEO (GSE181986).

### Immunohistochemistry (mouse).

PNs and normal sciatic nerves were dissected from *Dhh-Cre;Nf1^fl/fl^* mice and embedded in Tissue-Tek OCT compound (Thermo Fisher Scientific, 4585) and frozen on dry ice. Blocks were stored at –80°C until used. Cut sections (12 μm) were air-dried, fixed with 4% paraformaldehyde (PFA), and washed with Tris-buffered saline and 0.3% Tween 20 (TBST). The sections were blocked with 10% normal goat serum (NGS) in TBST for 30 minutes followed by incubation overnight at 4°C with primary antibodies against CD74 (Abcam, 245692; 1:100), CD11b (BD Pharmingen, 553308; 1:250), CD11c (Invitrogen, MA11C5; 1:500), and CNPase (Millipore, AB9342; 1:200). The next day, slides were washed in TBST 3 times and incubated in secondary antibodies at 1:500 (Alexa Fluor 594 [AF594] goat anti-rabbit, AF647 goat anti-rabbit, AF647 goat anti-rat, AF647 goat anti–Armenian hamster, and AF594 goat anti-chicken; Invitrogen). Sections were washed in TBST and incubated with DAPI (1:10,000) for 10 minutes. After coverslipping, fluorescence images were captured using a Zeiss Axiovert 200M immunofluorescence microscope with 405, FITC, Cy5, and Texas Red filters.

### Immunohistochemistry (human).

Human PNs were resected from NF1 patients undergoing debulking surgeries at the Center for Clinical Research of the NIH, fixed in 10% neutral buffered formalin solution (VWR) for 24 hours at room temperature, stored in 80% ethanol, and then embedded in paraffin. Cut sections (4 μm) were mounted to the microscope slides and stored at room temperature before use. Deparaffinized tissue sections were blocked in 1% peroxide in PBS for 15 minutes, blocked in PBS with normal serum for 1 hour at room temperature, and incubated with primary antibodies (anti-CD74, Sigma-Aldrich, HPA010592 [1:1600] and anti–NF-κB1, Sigma-Aldrich, HPA027305 [1:800]) at 4°C overnight. Tissue sections were washed with 0.1 M Tris-buffered saline with 0.1% Tween in TBS and incubated with secondary antibody conjugated with polymeric HRP for 1 hour at room temperature before staining with DAB for 2 minutes and counterstained with Mayer’s hematoxylin solution for 10 seconds. Stained slides were covered with glass cover slides in Permount and sealed with nail polish.

### Western blotting.

Nuclear extracts were loaded into 4%–20% Criterion TGX precast gels (Bio-Rad, 5671093). Proteins were transferred to PDVF membranes (Millipore, IPFL00010) and then blocked in 5% blotting-grade blocker (Bio-Rad, 1706404) in TBST, and then incubated with anti-p65 (Cell Signaling Technology, 4764; 1:1000) or anti-p50 (Delta Biolabs, DB035; 1:400) at 4°C overnight. Lamin B1 (Cell Signaling Technology, 12586; 1:2000) was used as loading control. Secondary HRP (1:10000) was added for 1 hour at room temperature prior to Immobilon Western Chemiluminescent HRP substrate (Millipore, WBKLS0500) and chemiluminescence imaging on an Azure Biosystems c500 ChemiDoc gel imager.

### Lentiviral preparations and transduction of SCPs.

Isolation and growth of SCP spheres from embryos and neurofibromas have been previously described (15, 24, 25). Dominant negative IκB-α S32A/S36A super suppressor (IκB-SS) and control plasmids have been described previously (44). For lentiviral preparation, 293T cells cultured in DMEM with 10% FBS were cotransfected with pMD.2 VSV-G envelope plasmid (psPAX2 helper plasmid) and IκB-SS or control plasmid DNAs with PEI added after 15 minutes. Virus was harvested 24 to 36 hours later and concentrated and purified with 20% sucrose. Secondary *Dhh-Cre;Nf1^fl/fl^* neurofibroma-derived spheres were transduced after plating in low-binding plates at a multiplicity of infection (MOI) of 1:50 for 4 days, and then sphere numbers were counted. E12.5 wild-type and *Nf1^–/–^* SCP spheres were infected at an MOI of 1:10 and counted after 5 days.

### Statistics.

A *P* value of less than 0.05 was considered significant unless otherwise specified. Parameters for scRNA-seq analyses are included in their respective methods and/or scripts available on Github (https://github.com/LKershner/neurofibroma).

### Study approval.

The animal care and use committee of Cincinnati Children’s Hospital Medical Center approved all animal care and procedures under IACUC protocol 2019-0018. Collection of human tissue was approved under NIH IRB protocol 10-C-0086.

## Author contributions

NS, JFS, and NR designed the research studies. JW and MP conducted experiments. LJK, KC, XZ, and MP acquired data. LJK, KC, XZ, MP, NS, and NR analyzed data. JFS and NR provided reagents. LJK, KC, XZ, NS, JFS, and NR wrote the manuscript.

## Supplementary Material

Supplemental data

Supplemental table 1

Supplemental table 2

Supplemental table 3

Supplemental table 4

Supplemental table 5

## Version 1 09/22/2022

Electronic publication
