# Supplementary material for: Multiple Nf1 Schwann cell populations reprogram the plexiform neurofibroma tumor microenvironment
Source: JCI Insight. 2022 Sep 22;7(18):e154513. doi: 10.1172/jci.insight.154513 (PMC9675562; doi:10.1172/jci.insight.154513)
Supplement: Supplemental data [file jciinsight-7-154513-s209.pdf]

SUPPLEMENTAL MATERIAL

Spreadsheet S1. scRNA-seq quality metrics of mouse PN (7 mo. *Dhh*-Cre;*Nf1*$^{fl/fl}$), pretumor (2 mo. *Dhh*-Cre;*Nf1*$^{fl/fl}$), and corresponding 7 mo. and 2 mo. *Nf1*$^{fl/fl}$ littermate controls as determined by Cell Ranger.

Spreadsheet S2. The top 50 markers for each cluster from the integrated mouse data object as determined by Seurat's FindAllMarkers function.

Spreadsheet S3. Seurat numerical cluster annotation, cell type annotation, cluster merging designations, and calculated doublet percentages.

Spreadsheet S4. Differentially expressed genes of plexiform versus seven month age-matched control and pretumor versus two month age-matched control. First two tabs show changes with a fold-change (FC) cutoff of >2. Last two tabs show all raw data.

Spreadsheet S5. CellPhoneDB predicted receptor-ligand interactions, shared between human and mouse PN and absent in mouse controls, for all clusters. Tab 1, Annotation key. Tab 2, Cluster interactions for each receptor ligand pair.

Fig. S1. Heatmap of top five markers for each cluster (Seurat). Shared markers shown once. For ease of visualization, each cluster was subsampled to 100 cells during heatmap generation.

Fig. S2. UMAP representation of doublet scores as determined by Scrublet, arrow shows cluster 25 which contained 55% doublets and was ignored for determining cell type proportions.

Fig. S3. DotPlot shows average expression of all transcripts encoding collagens in each cluster, highlighting the expression largely in fibroblast and SC clusters.

Fig. S4. Complete network plots for each SC cluster in PN, showing transcription factors up- or down-regulated versus controls (fold > 1.2), and genes predicted to be regulated by the transcription factors. Network plots for all clusters available as individual PDFs on Github.

Fig. S5. Integrated human PN UMAP showing original annotations, prior to mouse label transfer.

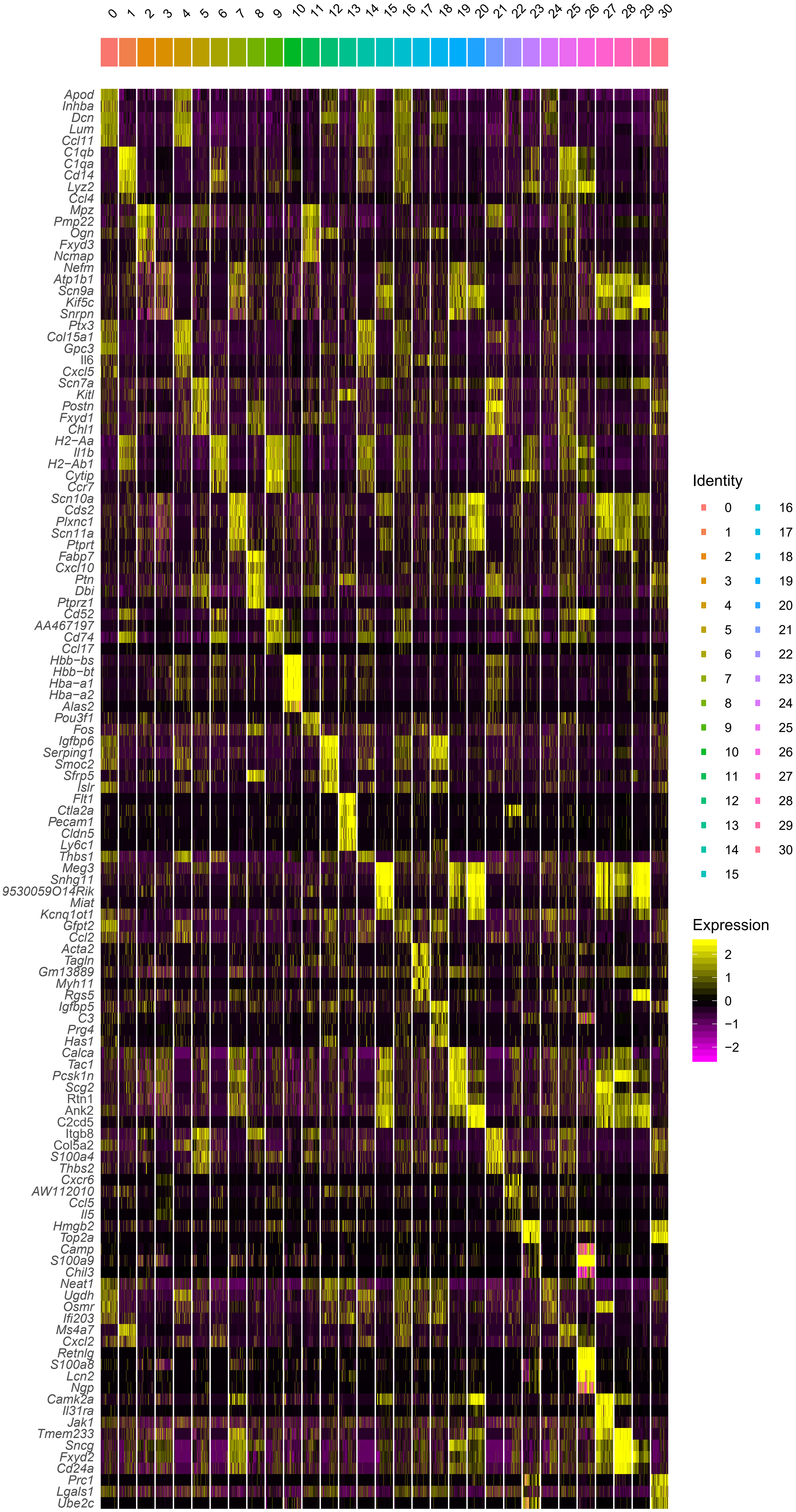

0 1 2 3 4 5 6 7 8 9 10 11 12 13 14 15 16 17 18 19 20 21 22 23 24 25 26 27 28 29 30
Apod
Inhba
Dcn
Lum
Ccl11
C1qb
C1qa
Cd14
Lyz2
Ccl4
Mpz
Pmp22
Ogn
Fxyd3
Ncmap
Nefm
Atp1b1
Scn9a
Kif5c
Snrpn
Ptx3
Col15a1
Gpc3
Il6
Cxcl5
Scn7a
Kitl
Postn
Fxyd1
Chl1
H2−Aa
Il1b
H2−Ab1
Cytip
Ccr7
Scn10a
Cds2
Plxnc1
Scn11a
Ptprt
Fabp7
Cxcl10
Ptn
Dbi
Ptprz1
Cd52
AA467197
Cd74
Ccl17
Hbb−bs
Hbb−bt
Hba−a1
Hba−a2
Alas2
Pou3f1
Fos
Igfbp6
Serping1
Smoc2
Sfrp5
Islr
Flt1
Ctla2a
Pecam1
Cldn5
Ly6c1
Thbs1
Meg3
Snhg11
9530059O14Rik
Miat
Kcnq1ot1
Gfpt2
Ccl2
Acta2
Tagln
Gm13889
Myh11
Rgs5
Igfbp5
C3
Prg4
Has1
Calca
Tac1
Pcsk1n
Scg2
Rtn1
Ank2
C2cd5
Itgb8
Col5a2
S100a4
Thbs2
Cxcr6
AW112010
Ccl5
Il5
Hmgb2
Top2a
Camp
S100a9
Chil3
Neat1
Ugdh
Osmr
Ifi203
Ms4a7
Cxcl2
Retnlg
S100a8
Lcn2
Ngp
Camk2a
Il31ra
Jak1
Tmem233
Sncg
Fxyd2
Cd24a
Prc1
Lgals1
Ube2c
Identity
0
1
2
3
4
5
6
7
8
9
10
11
12
13
14
15
16
17
18
19
20
21
22
23
24
25
26
27
28
29
30
Expression
2
1
0
−1
−2

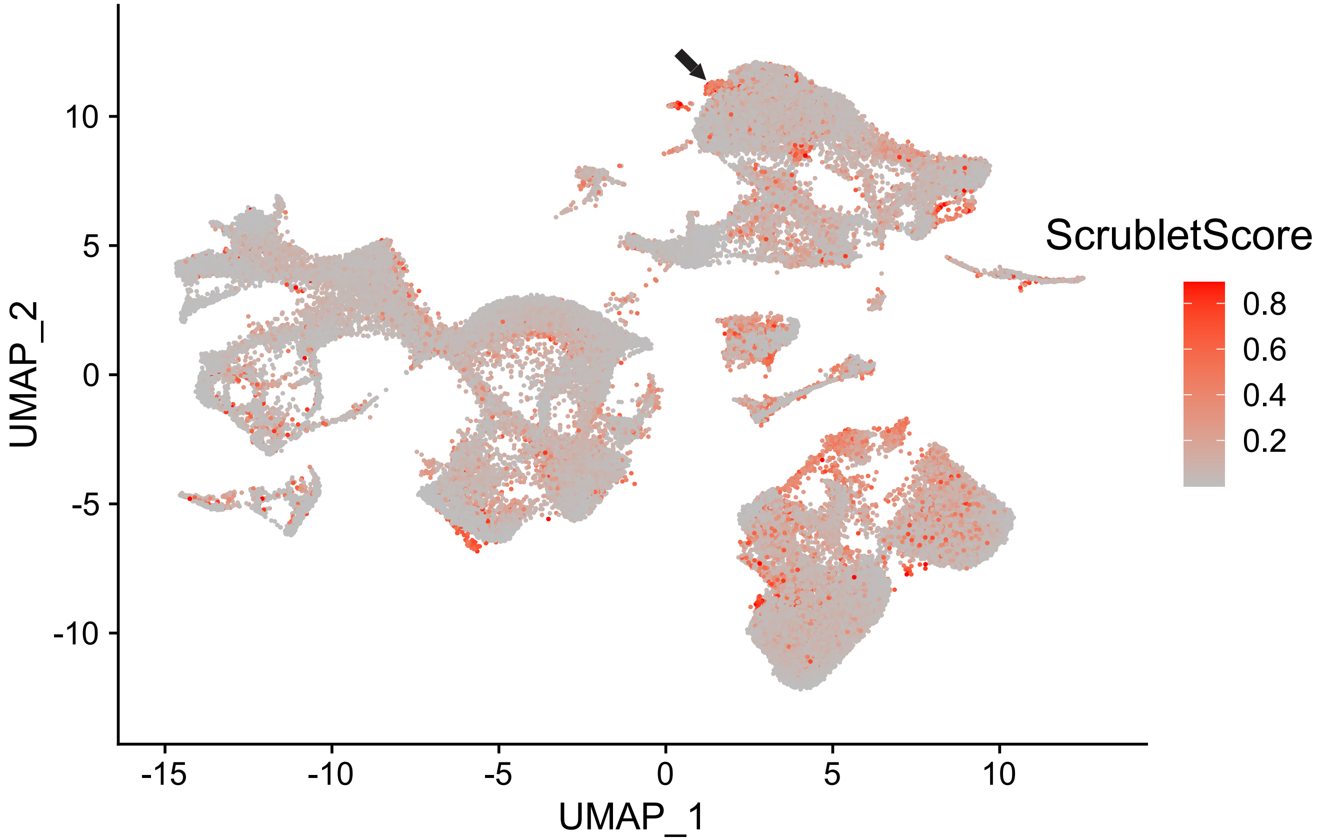

ScrubletScore
0.8
0.6
0.4
0.2
UMAP_2
10
5
0
-5
-10
-15
-10
-5
0
5
10
UMAP_1

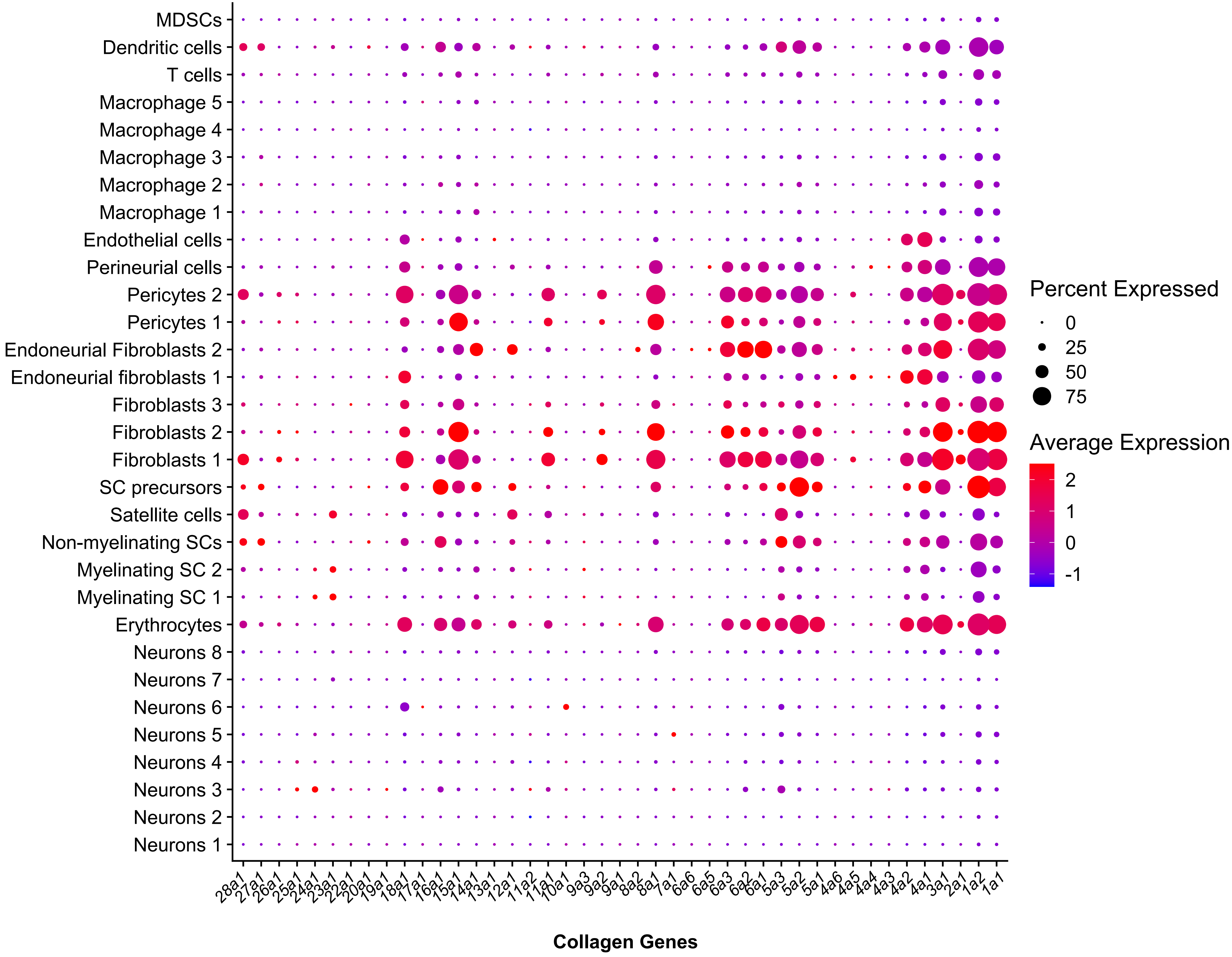

MDSCs
Dendritic cells
T cells
Macrophage 5
Macrophage 4
Macrophage 3
Macrophage 2
Macrophage 1
Endothelial cells
Perineurial cells
Pericytes 2
Pericytes 1
Endoneurial Fibroblasts 2
Endoneurial fibroblasts 1
Fibroblasts 3
Fibroblasts 2
Fibroblasts 1
SC precursors
Satellite cells
Non-myelinating SCs
Myelinating SC 2
Myelinating SC 1
Erythrocytes
Neurons 8
Neurons 7
Neurons 6
Neurons 5
Neurons 4
Neurons 3
Neurons 2
Neurons 1
28a1 27a1 26a1 25a1 24a1 23a1 22a1 20a1 19a1 18a1 17a1 16a1 15a1 14a1 13a1 12a1 11a2 11a1 10a1 9a3 9a2 9a1 8a2 8a1 7a1 6a6 6a5 6a3 6a2 6a1 5a3 5a2 5a1 4a6 4a5 4a4 4a3 4a2 4a1 3a1 2a1 1a2 1a1
Collagen Genes
Percent Expressed
0
25
50
75
Average Expression
2
1
0
-1

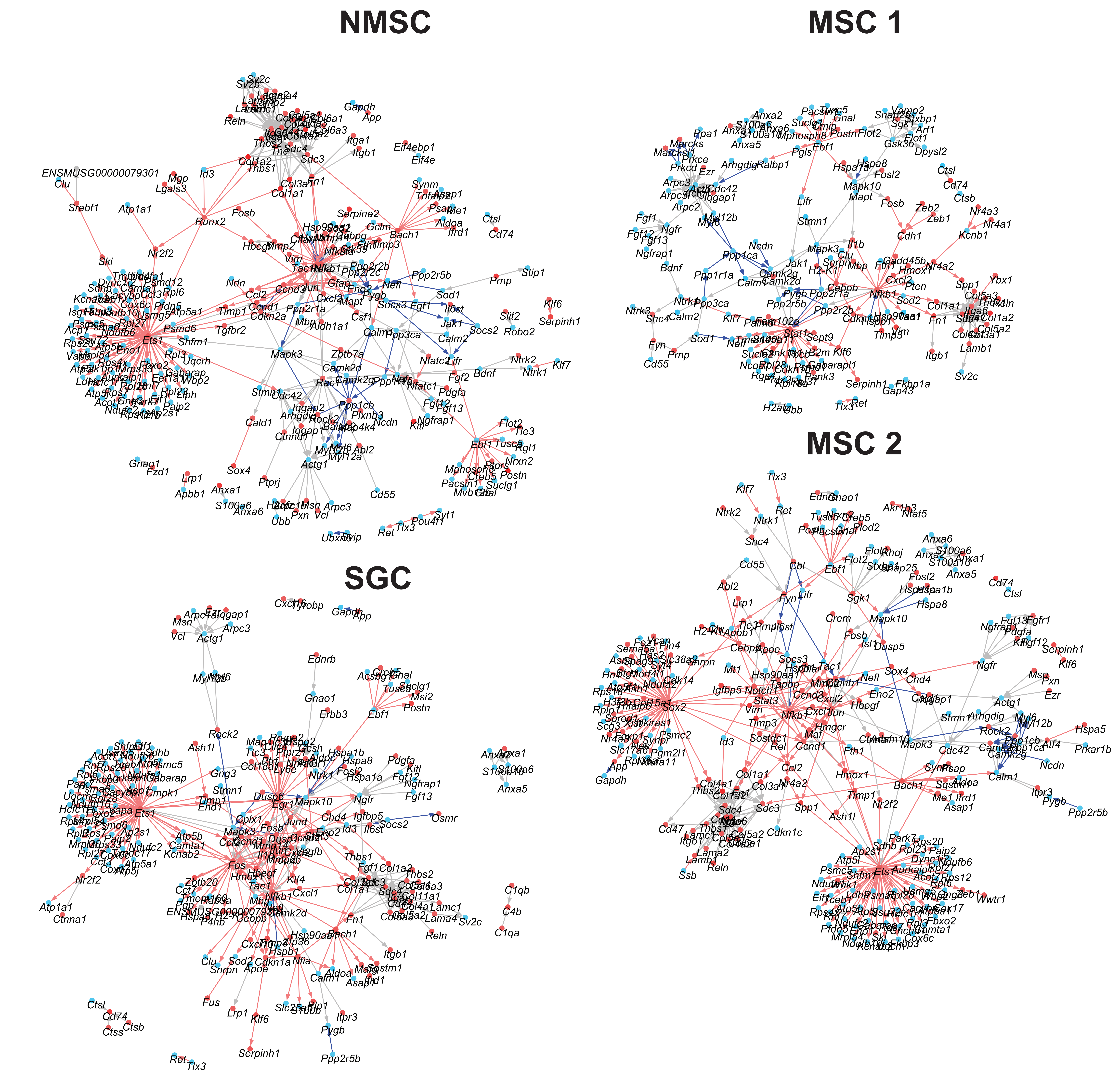
NMSC
MSC 1
MSC 2
SGC

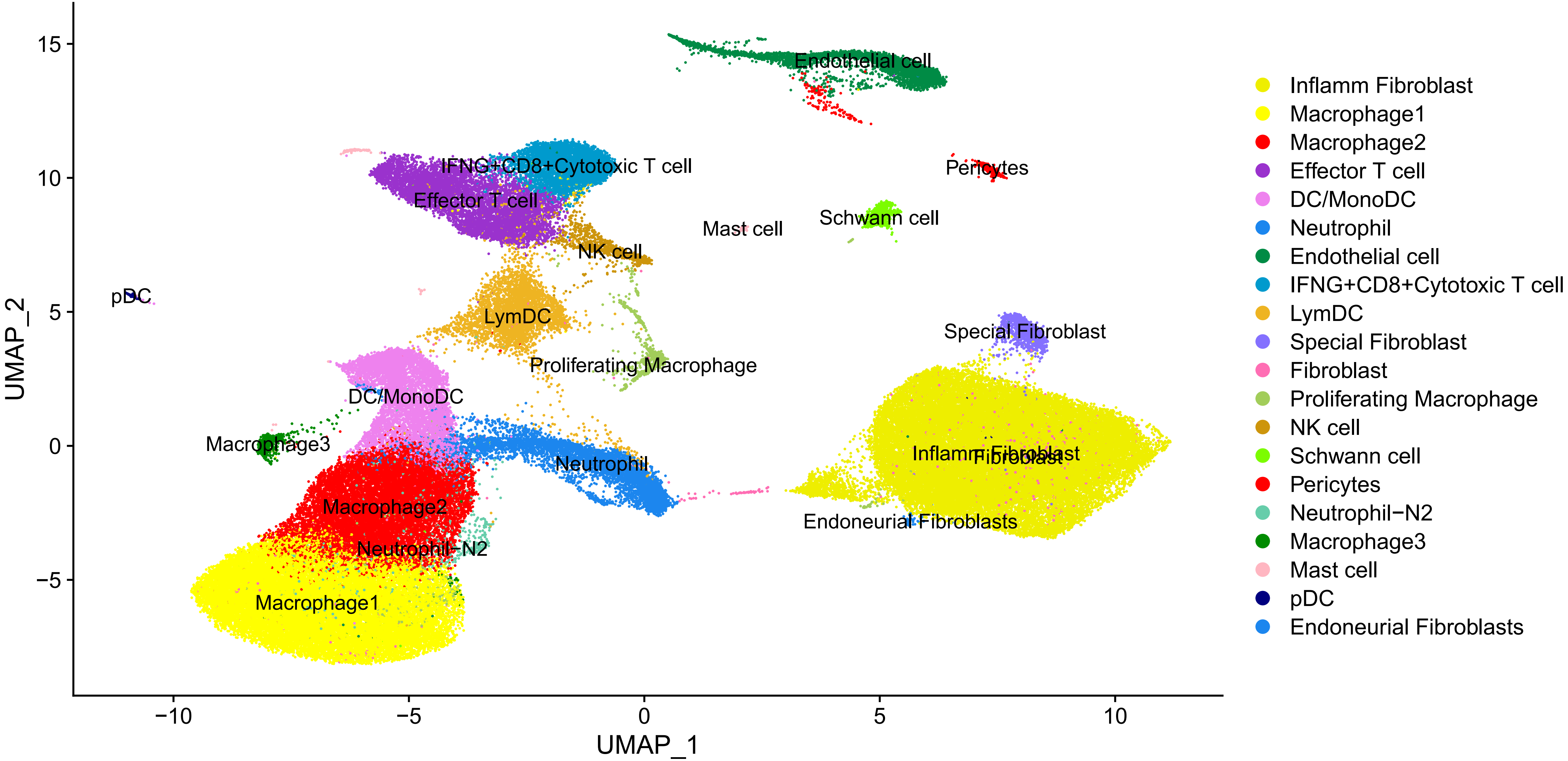

Endothelial cell
Pericytes
IFNG+CD8+Cytotoxic T cell
Effector T cell
Schwann cell
Mast cell
NK cell
pDC
LymDC
Special Fibroblast
Proliferating Macrophage
DC/MonoDC
Macrophage3
Neutrophil
Inflamm Fibroblast
Fibroblast
Endoneurial Fibroblasts
Macrophage2
Neutrophil−N2
Macrophage1
UMAP_2
UMAP_1
15
10
5
0
−5
−10
−5
0
5
10
Inflamm Fibroblast
Macrophage1
Macrophage2
Effector T cell
DC/MonoDC
Neutrophil
Endothelial cell
IFNG+CD8+Cytotoxic T cell
LymDC
Special Fibroblast
Fibroblast
Proliferating Macrophage
NK cell
Schwann cell
Pericytes
Neutrophil−N2
Macrophage3
Mast cell
pDC
Endoneurial Fibroblasts
